# Charge Polarity Control in Organic Transistors of Mixed and Segregated Complexes Based on Diaminonaphthalene and Pyrene

**DOI:** 10.1021/acsami.3c10583

**Published:** 2023-09-15

**Authors:** Nikhil Rao Mallela, Tadashi Kawamoto, Takehiko Mori

**Affiliations:** Department of Materials Science and Engineering, Tokyo Institute of Technology, O-okayama, Meguro-ku, Tokyo 152-8552, Japan

**Keywords:** organic transistors, charge-transfer complex, ambipolar transistor, mixed stack, single-crystal transistors

## Abstract

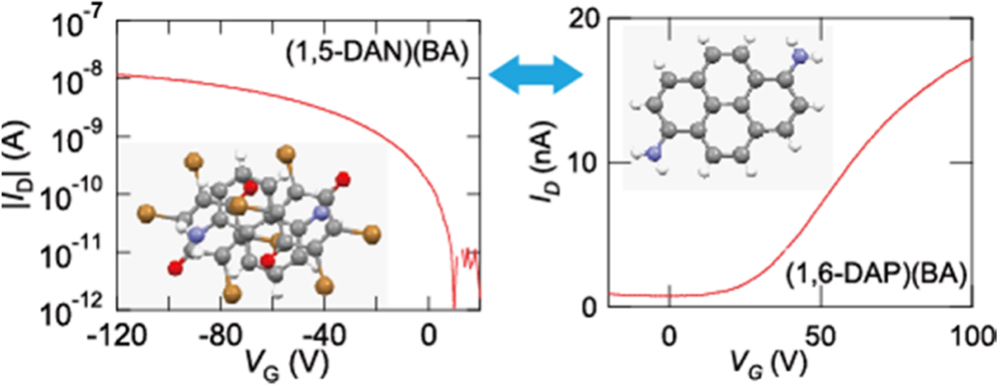

Organic cocrystals of diaminonaphthalene (DAN) and diaminopyrene (DAP) with bromanil (BA) and tetracyanoquinodimethane (TCNQ) are an exemplar system for understanding the charge-transport process, where from the viewpoint of partition theory, orbital symmetry plays a crucial role in controlling the carrier charge polarity of transistors. In the mixed-stack complexes of BA and other *p*-quinone acceptors, a comparatively weak donor, 1,5-DAN, shows p-channel characteristics owing to the counteractive contribution of the next highest occupied molecular orbital for electron transport. This characteristic behavior occurs because the BA molecule, situated on top of the amino group, overlaps with half of the DAN molecule. By contrast, the BA and TCNQ complexes of a stronger donor, 1,6-DAP, exhibit n-channel transport due to the cooperative path and orthogonal orbitals. Similarly, TCNQ complexes of variously substituted DAN show n-channel transport, where the TCNQ molecules are located on top of the DAN molecules. However, when the carbon electrodes of (1,5-DAN)(BA) are replaced by silver, electron transport appears due to the competitive effect of the Schottky barriers. In a highly conducting segregated complex of (1,6-DAP)(TCNQ), ambipolar transistor characteristics are observed without subtracting the bulk current by using carefully prepared thin-film transistors.

## 1 Introduction

Organic semiconductors have been studied for several decades due to their extraordinary chemical tunability, ease of processability, and versatile applications in flexible devices.^1,2^ Doping of organic semiconductors allows deliberate tuning of band alignment,^3^ operating voltage,^4^ and an increase in conductivity due to free carrier generation and has been proven advantageous in many organic electronic devices,^5^ including organic light-emitting diodes,^6^ field effect transistors,^7−9^ solar cells,^10^ and thermoelectrics.^11^ Organic doping results in charge transfer from an electron donor (D) to an electron acceptor (A). Studying the physical process of charge transfer and doping is crucial to understanding the charge transport in organic semiconductors. Charge-transfer complexes consisting of a highly ordered arrangement in either mixed stacks (–D–A–D–A–) or segregated stacks (–D–D–D– and –A–A–A–) are considered as a model to understand donor–acceptor systems.

Charge-transfer complexes are particularly characterized by a charge-transfer degree (ρ) ranging from neutral (ρ = 0) to ionic (ρ = 1), with the latter being comparatively scarce. As a result, most neutral complexes form mixed stacks that show semiconducting behavior. Although DA complexes contain both hole-transporting (D) and electron-transporting (A) units,^12−15^ most mixed-stack 1:1 crystals, particularly those of tetracyanoquinodimethane (TCNQ) complexes, show only electron (n-channel) transport.^16−24^ The Larsson partition theory explains this asymmetrical nature of charge transport in terms of effective (superexchange) transfer integrals *t*^eff^ by considering bridge orbitals other than the highest occupied molecular orbital (HOMO) and the lowest unoccupied molecular orbital (LUMO).^25−27^
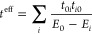
1The observed n-channel transport is related to the zero transfer integrals between D HOMO and A LUMO, due to the orthogonal overlap of these orbitals,^21,22^ wherein large transfers between D next HOMO and TCNQ LUMO mediate electron-only transport, and multiple nodes on A next LUMO lead to almost negligible transfer with D HOMO, impeding hole transport. We have previously explained the electron-only transport in TCNQ complexes of anthracene, tetracene, pyrene, and coronene along this line.^22,23^ This has been also demonstrated by the remarkably different bandwidths and effective masses in the conduction and valence bands.^28,29^ However, hole-only transport in mixed-stack complexes is strikingly rare.

Charge carrier polarity in transistors is also influenced by the charge injection barrier of the electrode. In a TCNQ complex of dibenzotetrathiafulvalene (DBTTF), the charge carrier changes from n-channel to ambipolar and finally to p-channel when the electrode potential deepens.^30^ TTF complexes of chloranil (CA) have been extensively studied due to the neutral-ionic transition,^31,32^ whereas transistor properties of CA and the analogues are scarcely reported. We have previously investigated p-channel characteristics in (tetramethylbenzidine)(CA). When CA in this complex is replaced by stronger acceptors, electron transport and bulk current gradually increase with an increase in ρ.^33^ A large ρ is also characteristic of segregated stacked DA cocrystals, which potentially exhibit ambipolar properties with high mobility. Balanced ambipolar transport has been reported in segregated complexes of (BEDT-TTF)(F_2_TCNQ) (ρ = 1) and (BEDT-TTF)(TCNQ) (ρ = 0.3) [BEDT-TTF: bis(ethylenedithio)-tetrathiafulvalene].^34,35^ However, realizing transistor properties in segregated stacks is challenging due to their high conductivity.

**Figure 1:**
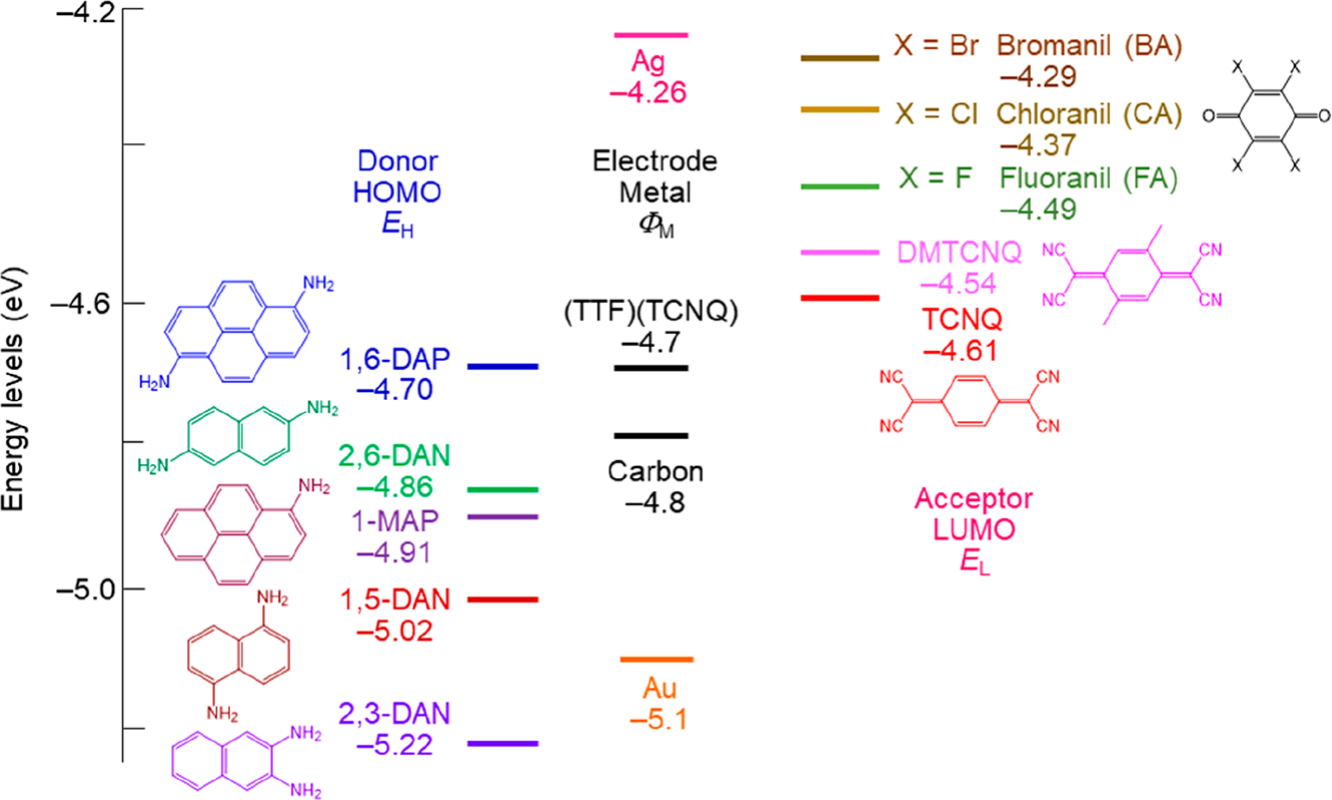
HOMO levels (eV) of diaminonaphthalene and diaminopyrenes together with the LUMO levels (eV) of the acceptors determined by the redox potentials assuming the reference energy level of ferrocene/ferrocenium to be –4.8 eV from the vacuum level (Figure S1).^36^

In the present work, we have investigated the transistor properties of charge-transfer complexes of diaminonaphthalene (DAN) and diaminopyrene (DAP). 1,6-DAP is a particularly strong donor (Figure 1); the TCNQ complex is practically ionic (ρ ∼ 1) and has segregated stacks.^37,38^ The conductivity is as high as 5 S cm^–1^, though the temperature dependence is semiconducting. Here, we show the transistor properties of this highly conducting segregated complex. The F_4_TCNQ and dimethyl-TCNQ (DMTCNQ) complexes are segregated as well,^39,40^ whereas the CA and bromanil (BA) complexes have mixed stacks.^41,42^ We have also investigated the structure and transistor properties of 1-aminopyrene (1-MAP) complexes.^43^

1,5-DAN is another sufficiently strong donor; in addition to the previously reported fluoranil (FA), CA, and BA complexes,^44^ we have newly obtained the TCNQ and DMTCNQ complexes. Furthermore, we have also examined complexes of other DAN isomers: 2,6-DAN and 2,3-DAN. Due to the low oxidation potentials of these donors, we can investigate the transistor properties without considering the hole transport limit of their energy levels.^45^

The transistor characteristics observed in these mixed-stack complexes are not balanced ambipolar. This unbalance of charge carrier polarity is explained by elucidating the significance of orbitals other than HOMO and LUMO from the viewpoint of Larsson partition theory.^21−27^ It is interesting to note that sometimes the observed charge polarity remarkably conflicts with the donor strengths because the superexchange transfers play a principal role.

Finally, to account for the influence of the electrode injection barrier in determining the charge carrier polarity of transistors,^30^ we have attempted to interpret the observed transistor properties by the combined use of the superexchange transfers and the charge injection barriers.

## 2 Experimental Section

### 2.1 Preparation and Identification of Cocrystals

The donor and acceptor molecules were individually dissolved in solvents, and the mixed solution was allowed to slowly evaporate at room temperature. Single crystals of (1,5-DAN)(FA), (1,5-DAN)(CA), (1,5-DAN)(BA),^44^ (2,6-DAN)(TCNQ), (2,6-DAN)(DMTCNQ), (2,3-DAN)(TCNQ), (1-MAP)(TCNQ),^43^ and (1-MAP)(DMTCNQ) were grown from toluene, while (1,5-DAN)(TCNQ), (1,5-DAN)(DMTCNQ), and (2,3-DAN)(DMTCNQ) were obtained from chlorobenzene. Crystals of (1,6-DAP)(BA) were grown from benzene.^41^ Microcrystals of (1,6-DAP)(TCNQ) and (1,6-DAP)(DMTCNQ) were grown from THF.^37^

The single-crystal X-ray data of (1,5-DAN)(TCNQ), (1,5-DAN)(DMTCNQ), (2,3-DAN)(TCNQ), (2,3-DAN)(DMTCNQ), and (1-MAP)(DMTCNQ) for structure analysis were taken by using a RIGAKU R-AXIS RAPID II imaging plate with Cu–Kα radiation from a rotation anode source with a confocal multilayer X-ray mirror (RIGAKU VM-spider, λ = 1.54187 Å). The structure was solved by the direct method (SHELXT) and refined by the full-matrix least-squares method by applying anisotropic temperature factors for all nonhydrogen atoms using the SHELXL programs (Table S2).^46,47^ The hydrogen atoms were placed at geometrically calculated positions. The crystal structures of (1,5-DAN)(FA) and (1,5-DAN) (BA) at 85 K were already reported,^44^ but the room-temperature (296 K) structures are included in the Supporting Information (Table S1).

For the measurements of charge-transfer absorption in thin films,^48^ the donor–acceptor complexes were dissolved in the parent solvent (0.01 wt %), filtered, and 20 μL of this solution was added dropwise on a glass substrate heated at 60 °C. After drying, the UV–vis–NIR absorption was measured on a JASCO V-670 spectrometer. The charge-transfer absorption was extracted after subtracting the background.

### 2.2 Device Fabrication

Doped n-type Si wafers with thermally grown SiO_2_ (300 nm, *C* = 11.5 nF/cm^2^) layers were used as substrates. The substrates were cleaned twice by sonication in ultrapure water, acetone, and isopropanol successively for 10 min each, followed by drying at 100 °C for 30 min and finally ultraviolet-ozone treatment for more than 20 min.

Cocrystals were grown on the surface of octyltrimethoxysilane (OTMS)-treated Si/SiO_2_ wafer by solvent-induced precipitation. Charge-transfer complexes were dissolved in the parent solvent (0.1 wt %), and 50 μL of this solution was dissolved in 950 μL of a poor solvent for the complexes (hexane for benzoquinone derivatives). Finally, a few droplets of this solution were drop-cast on the OTMS-treated substrates. This crystal growth method prevents incorporation of the solvent molecules in the final crystal.^49^ After about 1 h of drying at room temperature, cocrystals grown on the substrates were further dried under high vacuum (10^–3^ Pa) for 2 h to remove remnant solvent from the devices. Top-contact single-crystal transistors were completed by depositing droplets of carbon paste (DOTITE, XC-12) or silver paste on opposite sides of the crystal to ensure the crystal’s long axis was oriented in the channel direction.^23^ Since the crystals grew along the stacking axis, the transistor properties were measured along this axis. The devices were then allowed to dry for more than 30 min under ambient conditions.

Thin-film transistors were fabricated in a bottom-gate bottom-contact architecture. The bottom-contact electrodes were patterned by thermal deposition of (TTF)(TCNQ) through a shadow mask on a polystyrene (PS)-treated SiO_2_ substrates.^50^ Due to the lack of interfacial polarization, (TTF)(TCNQ) (−4.7 eV) shows low contact resistance both for hole and electron injection.^50,51^ Microcrystals of the complexes were then thermally deposited on the electrodes under a vacuum of 1 × 10^–3^ Pa.

All of the FET measurements were performed under vacuum of 10^–3^ Pa with a semiconductor parameter analyzer (Keithley 4200). The carrier mobilities were estimated from the linear and saturated regions.

### 2.3 Theoretical Calculations

Molecular orbital calculations in the B3LYP level with TZP basis set were performed by the Amsterdam Density Functional (ADF) program using the atomic coordinates taken from the room-temperature crystal structures without geometry optimization.^52^ In the triad method,^12,13^*t*_h_^eff^ was estimated from the splitting of the HOMO level of the two D molecules in the D–A–D triad. Similarly, *t*_e_^eff^ was estimated from the LUMO splitting of the A–D–A triad. In the partition method, *t*_e_^eff^ and *t*_h_^eff^ were obtained from eq 1.^21−23,25−27,45^ Here, for hole transport, 0 corresponded to the D HOMO and *i* was related to the various A orbitals. For electron transport, 0 was the A LUMO, and *i* came from the various D orbitals. The calculations of the DA dimer including coordinates of one D and one A molecules were used as energy levels of the complexes; *E*_0_ and *E*_*i*_ were taken from this calculation, and *t*_0*i*_ was estimated based on the frozen orbital approximation (Table S5).^22^

The simplified time-dependent density functional theory (sTD-DFT) calculations were carried out using the ADF program with the M06-2X functional and TZP basis set.^52^ The coordinates of the dimers were taken from the room-temperature crystal structures and used without geometry optimization. The calculated spectra were obtained by assuming a Lorentzian-type peak width of 0.1 eV.

## 3 Results

### 3.1 Energy Levels

Figure 1 shows energy levels of the donors and acceptors determined from the redox potentials.^36^ 1,6-DAP is a very strong donor with a low oxidation potential, which is even stronger than TTF (−4.78 eV).^53^ The HOMO level is as high as the LUMO level of TCNQ. DANs are moderately strong donors, though the donor ability considerably depends on the positions of the amino substitution (Figure S11); when the molecular orbital has a large amplitude at the amino-substituted carbon, the energy level increases significantly. Even 2,3-DAN (−5.22 eV) is a stronger donor than quarterthiophene (−5.3 eV).^22^ Such low oxidation potential allows us to exclude the possibility of hole transport being impeded due to the hole transport limit.^45^ 1-MAP is as strong as these DAN derivatives. Among these acceptors, *p*-quinones are slightly weaker acceptors than TCNQ, and the acceptor ability gradually decreases in the order of FA > CA > BA.

### 3.2 Crystal Structures

**Figure 2:**
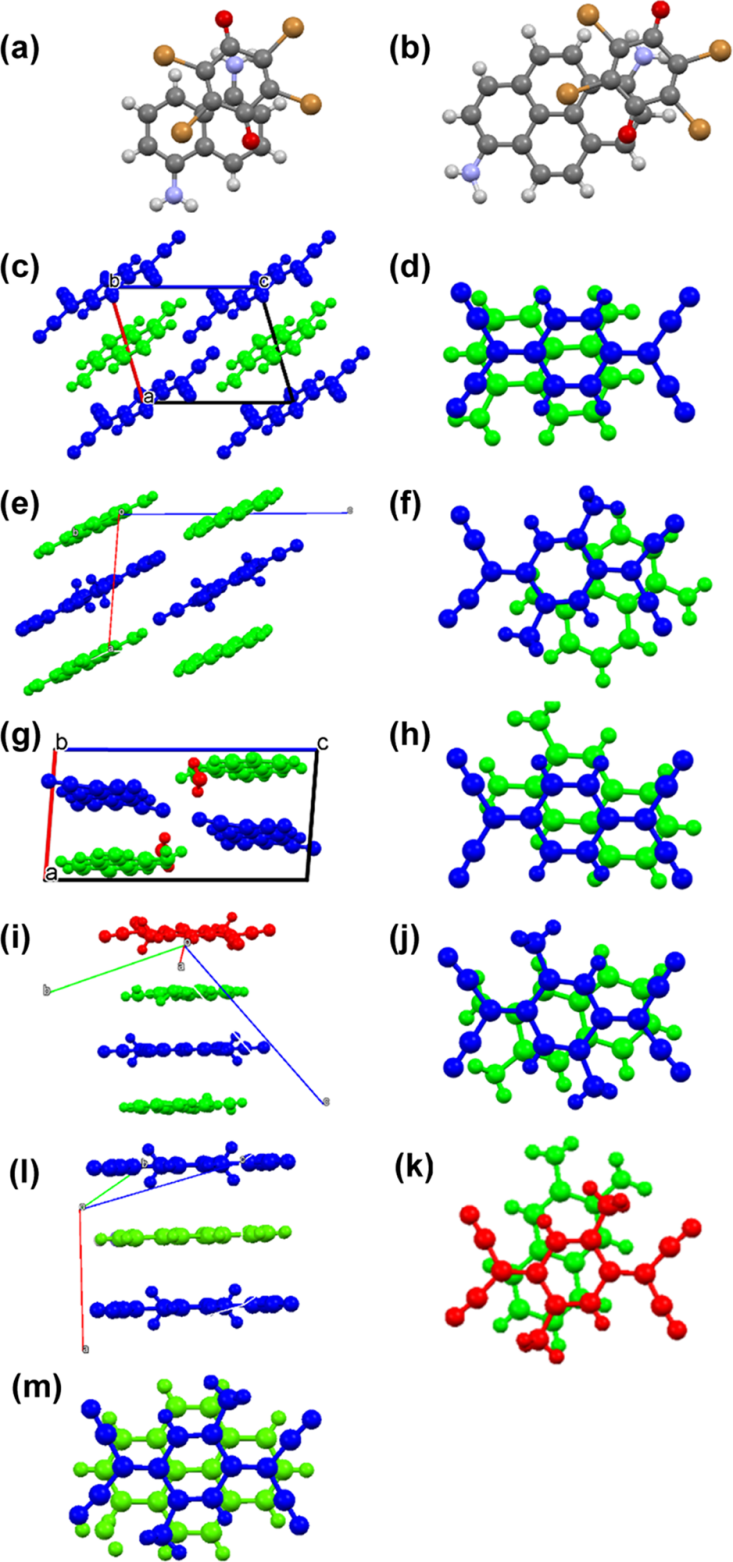
Overlap modes of (a) (1,5-DAN)(BA) and (b) δ-(1,6-DAP)(BA). Crystal structures and overlap modes of (c, d) (1,5-DAN)(TCNQ), (e, f) (1,5-DAN)(DMTCNQ), (g, h) (2,3-DAN)(TCNQ), (i, j, k) (2,3-DAN)(DMTCNQ), and (l, m) (1-MAP)(DMTCNQ), where the donor and acceptor molecules are designated in green and blue colors, respectively. In (g), water molecules are designated in red, and in (i, k), another crystallographically independent DMTCNQ molecule is in red.

It has been known that the FA, CA, and BA complexes of 1,5-DAN form mixed-stack crystals (Table S1).^44^ The acceptor phenyl rings are located on top of the amino groups (Figure 2a). A similar overlap pattern has been observed in δ-(1,6-DAP)(BA) (Figure 2b) and β-(1,6-DAP)(CA).^41,42^

We carried out single-crystal structure analyses of (1,5-DAN)(TCNQ), (1,5-DAN)(DMTCNQ), (2,3-DAN)(TCNQ), (2,3-DAN)(DMTCNQ), and (1-MAP)(DMTCNQ) (Table S2). These crystals align in mixed stacks as well. In (1,5-DAN)(TCNQ) (Figure 2c) and (1,5-DAN)(DMTCNQ) (Figure 2e), the donor and acceptor molecules are located on inversion centers and form mixed stacks along the crystallographic *a* axis with interplanar spacings of 3.24 and 3.36 Å, respectively. In contrast to the BA complexes, the TCNQ rings are located on top of the naphthalene rings, though the molecules are somewhat rotated (Figure 2d,f). We could not determine crystal structures of (2,6-DAN)(TCNQ), (2,6-DAN)(DMTCNQ), and (1,6-DAP)(DMTCNQ).

(2,3-DAN)(TCNQ) comprises water molecules between the stacks (Figure 2g). The 2,3-DAN and TCNQ molecules are located in general positions and form mixed stacks along the crystallographic *a* axis with an interplanar spacing of 3.32 Å. In (2,3-DAN)(DMTCNQ), the 2,3-DAN molecule is again located in a general position, but the DMTCNQ molecule is situated on an inversion center (Figure 2i). Accordingly, there are two crystallographically independent DMTCNQ molecules, which are rotated by 18 and 72° from the long axis of the 2,3-DAN molecule (Figure 2j,k). These TCNQ molecules alternately form a mixed stack in a DADA’ manner along the crystallographic *a* + *b* + *c* axis with interplanar spacings of 3.36 and 3.47 (Figure 2e).

Similar to (1-MAP)(TCNQ),^43^ (1-MAP)(DMTCNQ) has mixed stacks with an interplanar spacing of 3.45 Å (Figure 2l,m). The TCNQ and DMTCNQ molecules are located right on top of the donor molecule, and the molecules are perpendicular to the stacking (*a*) axis (Figure 2l). In (1-MAP)(TCNQ), the orientations of the amino groups are ordered within a stack, and there are two oppositely orientated stacks. However, in (1-MAP)(DMTCNQ), the amino orientation is disordered in a stack.

For the TCNQ and DMTCNQ complexes of various DAN, charge-transfer degrees ρ estimated from the bond lengths are nearly zero, but ρ from the infrared stretching modes are around 0.3 (Table S3). The values are comparatively large for mixed-stack complexes but reasonable in view of the moderately strong donor ability. The ρ values of the CA and BA complexes of 1,5-DAN have been reported to be 0.275 and 0.23 from infrared, respectively,^41,42^ while those for the 1-MAP complexes are about 0.2–0.3 from the infrared peaks and bond lengths.

### 3.3 Charge-Transfer Absorption

**Figure 3:**
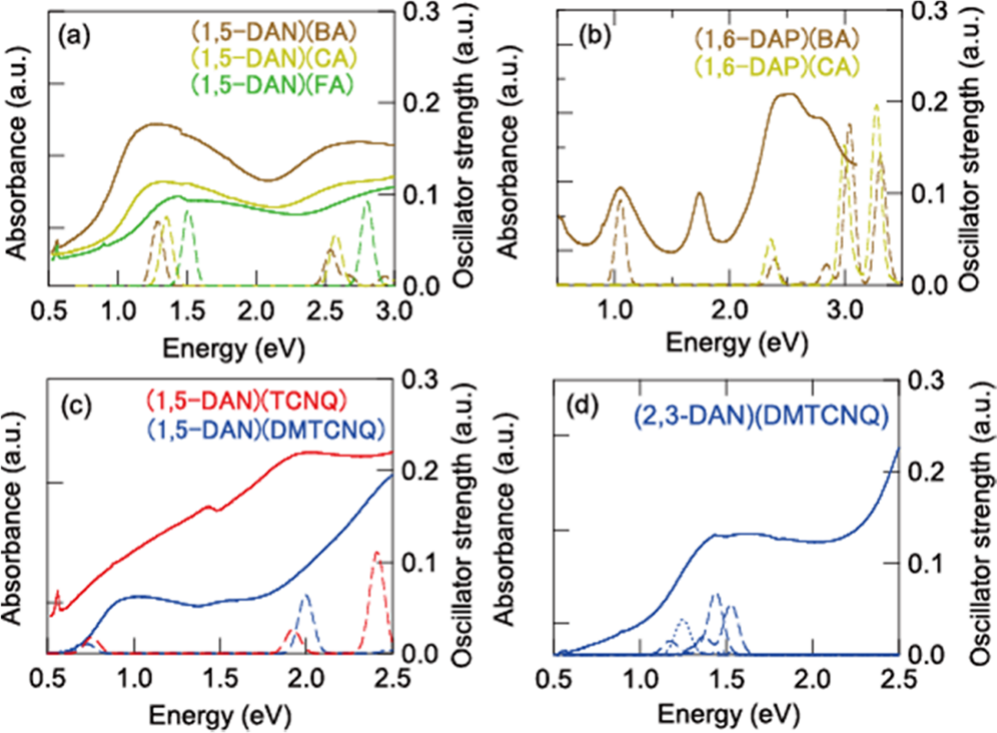
Thin-film charge-transfer absorption spectra of (a) 1,5-DAN complexes of *p*-benzoquinone derivatives, (b) (1,6-DAP)(BA), (c) TCNQ complexes of 1,5-DAN, and (d) (2,3-DAN)(DMTCNQ), together with calculated oscillator strengths (dashed curves). (2,3-DAN)(DMTCNQ) has two types of DA overlaps.

It has been demonstrated that when the donor HOMO and the acceptor LUMO are orthogonal, the charge-transfer absorption does not correspond to the HOMO/LUMO transition, rather corresponds to the transition from the next HOMO (HOMO-1) to LUMO.^48^ Thus, charge-transfer absorption of the thin films was investigated (Figure 3). The oscillator strengths calculated by sTD-DFT are plotted in dashed curves.

Complexes of 1,5-DAN and *p*-benzoquinones show two broad peaks at around 1.3 and 2.7 eV (Figure 3a). These peaks are respectively in good agreement with the calculated HOMO/LUMO and HOMO-1/LUMO transitions, indicating that these complexes have nonorthogonal HOMO/LUMO overlaps. The observed transition increases in the order of BA < CA < FA; this is opposite to the acceptor ability (Figure 1). The sTD-DFT calculation, however, reproduces the observed order; the HOMO/LUMO transfer integral increases in this sequence (Figure 1 and Table S5). In general, the observed charge-transfer transitions are well correlated with the calculated D HOMO/A LUMO gaps of the complexes rather than the difference in donor and acceptor potentials (Figure S2).

Due to the improved donor ability of 1,6-DAP, a HOMO/LUMO transition of (1,6-DAP)(BA) is expected below 1 eV (Figure 3b), but less obvious than Figure 3a. Figure 3b also demonstrates the absence of the HOMO/LUMO transition in the calculation based on α-(1,6-DAP)(CA). In (1,5-DAN)(TCNQ) and (1,5-DAN)(DMTCNQ) (Figure 3c), the HOMO/LUMO transition less than 1 eV is obscure due to the practical orthogonality (Table S5), but the HOMO-1/LUMO transition appears around 2 eV. The HOMO/LUMO transition of (2,3-DAN)(DMTCNQ) emerges around 1.5 eV due to the comparatively weak donor ability (Figure 3d). Interestingly, the HOMO-1/LUMO transition appears around the same energy as the HOMO/LUMO transition due to the approximate degeneracy of the HOMO and HOMO-1 levels (Figure S11a).

### 3.4 Transistor Properties of Mixed-Stack Complexes

**Figure 4:**
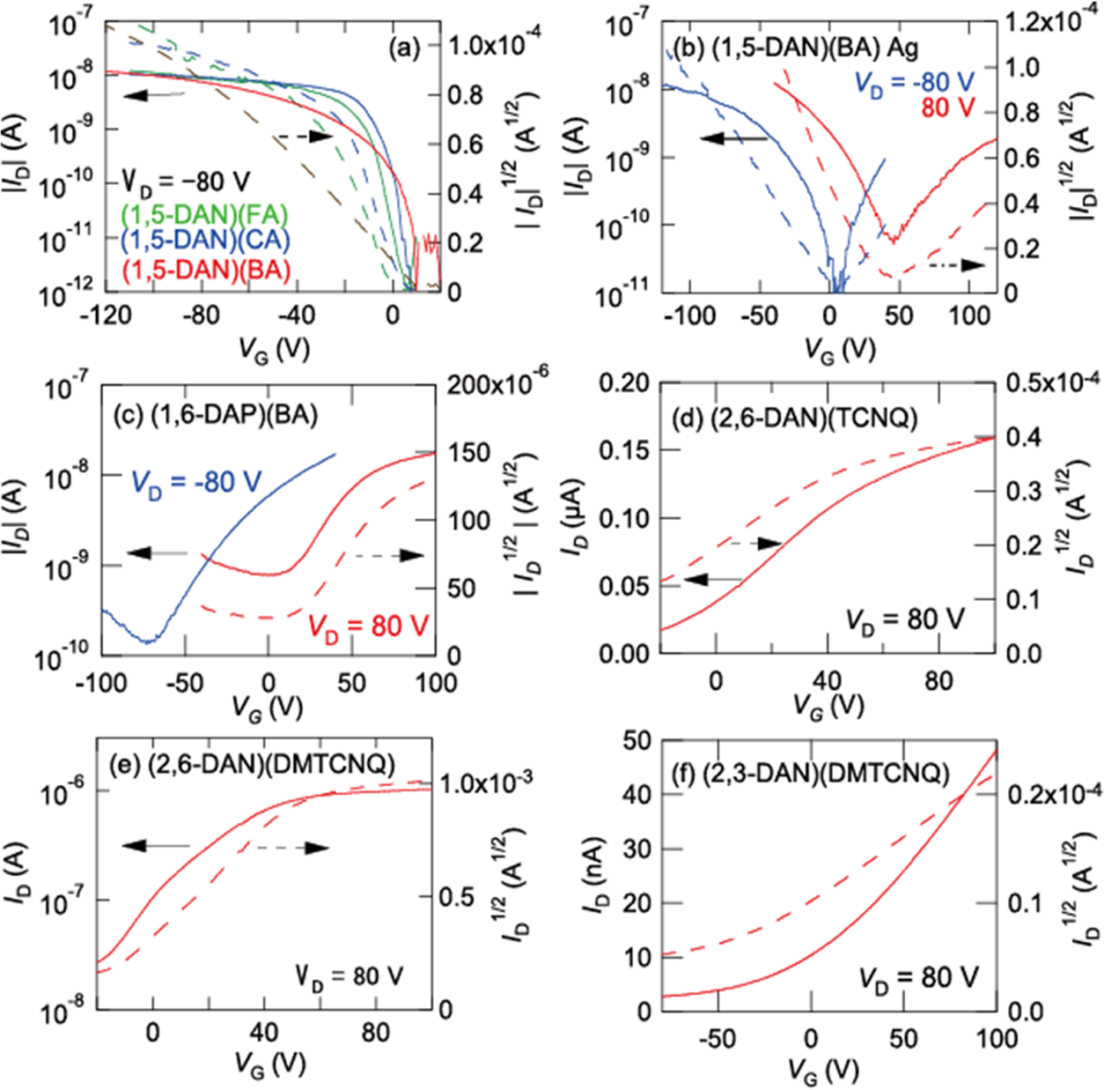
Transfer characteristics of single-crystal transistors of (a) (1,5-DAN)(FA), (1,5-DAN)(CA), and (1,5-DAN)(BA) with carbon electrodes and (b) those of (1,5-DAN)(BA) with silver electrodes. Transfer characteristics of thin-film transistors of (c) (1,6-DAP)(BA), (d) (2,6-DAN)(TCNQ), (e) (2,6-DAN)(DMTCNQ), and a single-crystal transistor of (f) (2,3-DAN)(DMTCNQ).

FA, CA, and BA complexes of 1,5-DAN show p-channel characteristics (Figure 4a). Naively, the p-channel characteristics are reasonable in view of the comparatively low reduction potential of these acceptors (Figure 1); among the tetramethylbenzidine complexes, only the CA complex shows p-channel characteristics as well.^33^ With silver electrodes, however, (1,5-DAN)(BA) shows ambipolar characteristics (Figure 4b). The hole mobility μ_h_ is still several times larger than the electron mobility μ_e_; the transistor parameters are extracted in Table 1.

**Table 1:** Transistor Parameters of the Complexes

complex	fabrication method^a^	μ_ave_ [μ_max_] (cm^2^ V^–1^ s^–1^)	*V*_T_ (V)	*I*_on_/*I*_off_
(1,5-DAN)(FA)	SC C	h 8.9 × 10^–4^ [2.2 × 10^–3^]	15^b^	8 × 10^3^
(1,5-DAN)(CA)	SC C	h 6.8 × 10^–4^ [2.4 × 10^–3^]	10^b^	1 × 10^4^
(1,5-DAN)(BA)	SC C	h 1.1 × 10^–3^ [2.0 × 10^–3^]	6^b^	3 × 10^4^
	SC Ag	e 3.4 × 10^–4^ [1.8 × 10^–3^]^c^	40	30
		h 1.3 × 10^–3^ [8.3 × 10^–3^]^c^	6	3 × 10^3^
(1,6-DAP)(BA)	Ev Q BC	e 1.2 × 10^–4^ [1.4 × 10^–4^]	2	20
(1,5-DAN)(TCNQ)	SC C	e 2.3 × 10^–8^	15	1.04
(1,5-DAN)(DMTCNQ)	SC C	e 7.3 × 10^–7^	10	1.17
(2,6-DAN)(TCNQ)	Ev Q BC	e 5.4 × 10^–3^	–30	8
(2,6-DAN)(DMTCNQ)	Ev Q BC	e 3.4 × 10^–4^	–20	40
(2,3-DAN)(DMTCNQ)	SC C	e 3.9 × 10^–5^	–65	16
(1-MAP)(TCNQ)	Ev Q	e 4.4 × 10^–4^ [5.3 × 10^–4^]	–20	1.7
(1,6-DAP)(TCNQ)	Ev Q	e 1.1 × 10^–3^ [1.3 × 10^–3^]^d^	–	–
		h 8.1 × 10^–4^ [1.1 × 10^–3^]^d^		
	Ev Q BC	e 1.4 × 10^–4^ [5.2 × 10^–4^]	–25	6
		h 1.1 × 10^–4^ [3.6 × 10^–4^]	20	4
(1,6-DAP)(DMTCNQ)	Ev Q	e 1.2 × 10^–4^ [1.4 × 10^–4^]	–30	50
		h 4.4 × 10^–4^ [5.3 × 10^–4^]	–15	1.5 × 10^2^

a SC: single crystal, Ev: evaporated thin film, C: carbon, Q: (TTF)(TCNQ), and BC: bottom contact (otherwise top contact).

b Subthreshold swing is 6, 4, and 7 V/decade for the FA, CA, and BA complexes, respectively.

c h 5.4 × 10^–4^ [1.8 × 10^–3^] and e 2.1 × 10^–4^ [1.8 × 10^–3^] in air.

d After subtracting the bulk current.

By contrast, (1,6-DAP)(BA) exhibits electron-dominant ambipolar characteristics (Figure 4c); although the transfer characteristics exhibit mainly electron transport, hole transport is obvious in the output characteristics in some samples (Figure S6b). This is surprising because 1,6-DAP is a much stronger donor than 1,5-DAN (Figure 1). Transistor properties are not observed in (1,6-DAP)(CA).

The TCNQ and DMTCNQ complexes of 1,5-DAN and 2,6-DAN show mainly n-channel transport (Figures 4d,e, S5a and S5b). (2,3-DAN)(TCNQ) does not show transistor properties probably due to the presence of water molecules. (2,3-DAN)(DMTCNQ) and (1-MAP)(TCNQ) again show mainly n-channel characteristics (Figures 4f and S5c). Since ρ for these complexes is not sufficiently small (Table S3), comparatively large off-current is unavoidable.^33^

### 3.5 Transistor Properties of Segregated-Stack Complexes

**Figure 5:**
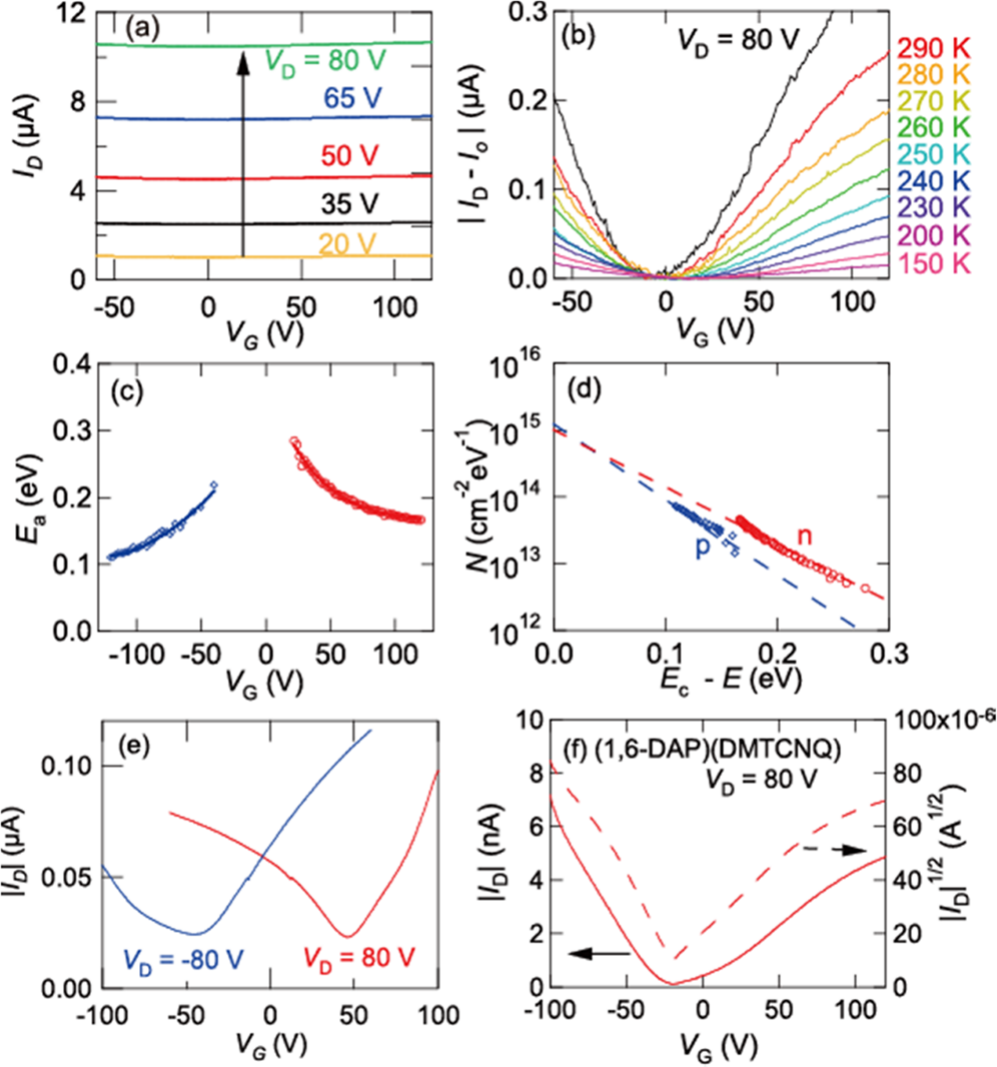
(a) Transfer characteristics of a thin-film (50 nm on PS) transistor of (1,6-DAP)(TCNQ) with top-contact (TTF)(TCNQ) electrodes. (b) Transfer characteristics after subtracting the bulk current at various temperatures. (c) *V*_G_ dependence of *E*_a_. (d) Trap density of states *N*(*E*). (e) Transfer characteristics of a thin-film (30 nm on PS) transistor of (1,6-DAP)(TCNQ) with bottom-contact (TTF)(TCNQ) electrodes. (f) Transfer characteristics of a thin-film (50 nm on OTMS) transistor of (1,6-DAP)(DMTCNQ) with top-contact (TTF)(TCNQ) electrodes.

An evaporated film (50 nm) of (1,6-DAP)(TCNQ) exhibited a room-temperature conductivity of σ_rt_ = 0.008 S cm^–1^ and an activation energy of *E*_a_ = 0.17 eV (Figure 5a). These values are reasonable in comparison with the reported single-crystal values, σ_rt_ = 5 S cm^–1^ and *E*_a_ = 0.26 eV.^37^ The *V*_D_ – *I*_D_ characteristics show *V*_D_^2^ dependence (Figure S7), suggesting the space-charge-limited-current nature. Although the gate voltage dependence is small (Figure 5a), after the bulk current *I*_0_ is subtracted, the thin-film transistors show balanced ambipolar properties (Figure 5b), where the gate modulation is 2% of the bulk current. The extracted mobilities and other parameters are listed in Table 1. The transfer integrals in the donor and acceptor stacks are similar (188 and 213 meV in Table S4); hence, balanced ambipolar characteristics are reasonable. In analogy with indigo derivatives,^54^ the interstack transfer integrals are as large as one-third (∼60 meV) of the intrastack transfer integrals due to the hydrogen bonds (Table S4). This is reminiscent of the reported small anisotropy of the single-crystal conductivity and the small difference between the powder and single-crystal conductivity.^37^ It is crucial to use active layer films as thin as possible, yet despite the high conductivity, we can extrapolate the transistor properties after subtracting the large bulk current.

With decreasing temperature, *I*_*D*_ – *I*_0_ decreases (Figure 5b), suggesting hopping transport. Activation energy, *E*_a_, evaluated by the Arrhenius plot of *I*_D_ – *I*_0_ at respective *V*_G_ (Figure 5c) is inversely proportional to *V*_G_ – *V*_T_,^55^ where the values are in the same order as the bulk *E*_a_ (0.17 eV). The trap density of states is obtained using *N*(*E*) = *C*/*q*(d*V*_G_/d*E*_a_),^56^ where *C* is the capacitance and *q* is the elemental charge. Since Figure 5d is well represented by straight lines, *N*(*E*) follows an exponential distribution, *N*(*E*) = *N*_G_ exp ((*E* – *E*_c_)/*kT*_G_), where *E* – *E*_c_ is the energy measured from the conduction band edge *E*_c_.^55^ Here, the parameters are *N*_G_ = 1.0/1.6 × 10^15^ cm^2^ eV^–1^ and *T*_G_ = 530/480 K for electron/hole. Accordingly, the total trap numbers are *qN*_G_*kT*_G_/*C* = 990/1380 V in units of *V*_G_.

Since dimerization has not been observed even at low temperatures,^37^ the semiconducting behavior has been attributed to the Mott insulating state. The balanced ambipolar properties are possibly related to the Mott insulating state, as well. Although the κ-type organic conductors are regarded as Mott insulators, the observed field effect transport is not symmetrical.^57^ (BEDT-TTF)(TCNQ) shows comparatively balanced electron/hole transport.^35^ The present observations have some similarities to (BEDT-TTF)(F_2_TCNQ) with an ionic character.^34^ The symmetrical electron–hole transport is potentially associated with the Mott insulating state coming from the ionic state.

When the top-contact (TTF)(TCNQ) electrodes are replaced with bottom-contact (TTF)(TCNQ) electrodes, we have observed satisfactory transfer characteristics without subtracting the bulk current (Figure 5e). For (1,6-DAP)(DMTCNQ), sufficient transfer characteristics are observed even in the top contact (Figure 5f). Although ρ is also practically one as well (Table S2), the slightly weaker acceptor ability leads to a smaller bulk conductivity.

## 4 Discussion

### 4.1 Effective Transfer Integrals

Effective transfer integrals are calculated by the triad and partition methods, as listed in Table 2. The typical values are usually around 50–70 meV. Since transfer integrals of single-component organic semiconductors as well as segregated stacks (Table S4) are in the order of 100–250 meV,^58,59^ the superexchange transfers are typically one-third of the direct transfers.

**Table 2:** Energy Levels and Effective Transfer Integrals (meV)

					triad	partition
complex	D HOMO^a^ (eV)	A LUMO^a^ (eV)	ρ^b^	*t*_DA_	*t*_e_^eff^/ *t*_h_^eff^	*t*_e_^eff^/ *t*_h_^eff^
(1,5-DAN)(FA)	–4.99 (−5.02)	–4.90 (−4.49)		382	45/58	3/62
(1,5-DAN)(CA)^c^	–5.00 (−5.02)	–4.60 (−4.37)	0.275^43^	325	40/54	13/57
(1,5-DAN)(BA)	–4.95 (−5.02)	–4.56 (−4.29)	0.23^43^	315	42/45	12/58
δ-(1,6-DAP)(BA)	–4.73 (−4.70)	–4.60 (−4.29)	0.16^41^	275	61/60	10/30
α-(1,6-DAP)(CA)	–4.68 (−4.70)	–4.44 (−4.37)	∼0^42^	7	43/6	9/1
β-(1,6-DAP)(CA)	–4.68 (−4.70)	–4.44 (−4.37)	∼0^42^	329	67/79	16/63
(1,5-DAN)(TCNQ)^c^	–4.87 (−5.02)	–5.20 (−4.61)	0.31 (0.06)	162	52/27	73/20
(1,5-DAN)(DMTCNQ)	–4.99 (−5.02)	–4.86 (−4.54)	0.38 (0.00)	129	3/9	40/4
(2,3-DAN)(DMTCNQ)	–5.33 (−5.22)	–4.87 (−4.54)	0.33 (0.01)	153	63/24,20	22/37,19
(1-MAP)(TCNQ)	–4.85 (−4.91)	–5.22 (−4.61)	0.23 (0.25)	117	63/18	68/4
(1-MAP)(DMTCNQ)	–4.96 (−4.91)	–4.91 (−4.54)	0.36	75	37/6	58/1

a Calculated energy levels of the individual molecules. Values in the parentheses (Figure 1) are from the cyclic voltammetry (Figure S1).^36^

b Charge-transfer degree from infrared. Values from the bond lengths are in parentheses (Table S3).

c Based on the crystal structures at 85 K for (1,5-DAN)(CA) and 106 K for (1,5-DAN)(TCNQ). Others are from room-temperature crystal structures.

**Figure 6:**
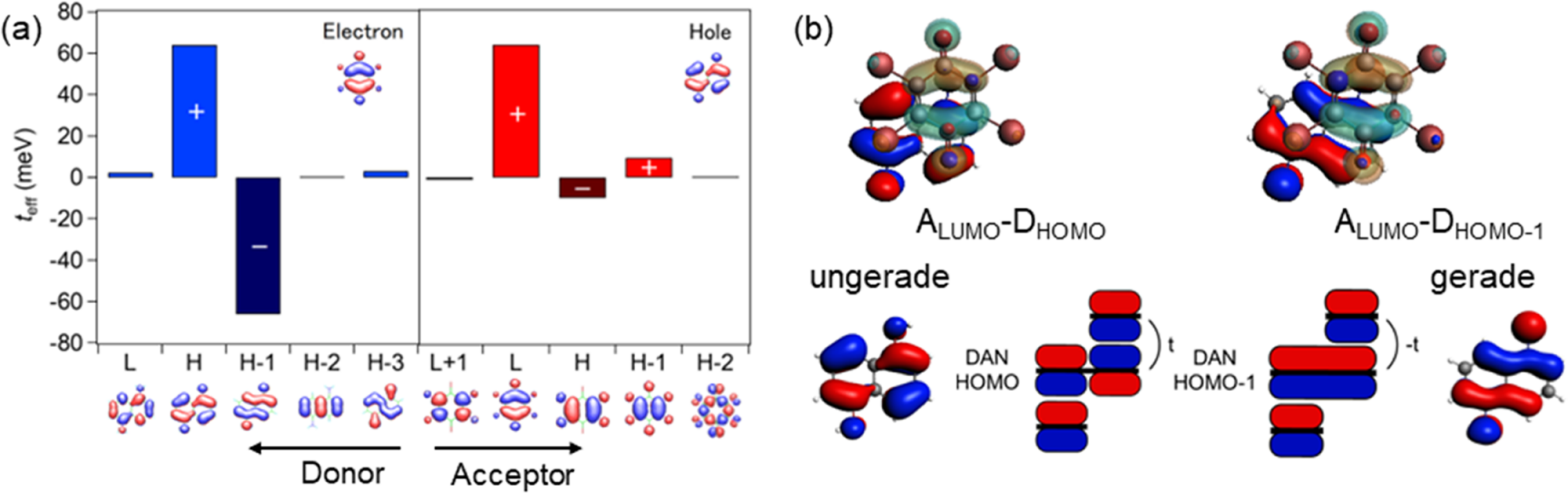
(a) Contributions in the partition method in (1,5-DAN)(BA). (b) Orbital overlap of HOMO and HOMO-1.

For the (1,5-DAN)(BA) series complexes, the effective transfer integrals calculated by the partition method are largely unbalanced, suggesting hole-dominant transport (Table 2). This is in agreement with the observed p-channel transistor properties (Figure 4a). The detailed investigation of the respective orbitals’ contribution (Table S5) reveals that for electron path, the D HOMO contribution is mostly canceled by the HOMO-1 contribution (Figure 6a). As shown in Figure 6b, the A LUMO is overlapped with the (right) half of 1,5-DAN, where the HOMO and HOMO-1 are antibonding and bonding combinations of approximately the same orbital. Since the antibonding and bonding combinations have gerade and ungerade symmetry, these transfers result in opposite-sign contributions in eq 1 and thus cancel each other. Consequently, the effective electron transfer is significantly smaller than the effective hole transfer. This unbalance is not obvious in the triad method (Table 2) and energy band calculations.^60^ In the triad ADA calculation, the HOMO and HOMO-1 are modified when hybridized with the A LUMO, and the actual bridging orbitals are different from those of the D HOMO and HOMO-1. Accordingly, the exact cancellation does not occur.^61^ Similar hole-dominant transport has been observed in quarterthiophene (4T) complexes of TCNQ,^22^ where the TCNQ molecule overlaps with half of 4T as well. Such hole-dominant transport is characteristic of the half-molecular overlap along with multiple nonorthogonal orbitals.

In (1,6-DAP)(BA), two polymorphs have been reported;^41^ in the δ-form, the BA molecule is located on the top of an amino group (Figure 2b). Consequently, this phase is also hole-dominant (*t*_h_^eff^ > *t*_e_^eff^) according to the same mechanism as that of (1,5-DAN)(BA) (Table S5d). In the γ-form, however, the BA molecule is located exactly on the top of the 1,6-DAP molecule, but the atomic coordinates have not been reported. Hence, the CA complexes are investigated to understand the charge carrier asymmetry. In the α-form,^42^ the CA molecule is located on the top of the 1,6-DAP molecule, and the A LUMO and the D HOMO are orthogonal. As a consequence, *t*_e_^eff^ is much larger than *t*_h_^eff^ even in the triad method (Table 2). However, the CA molecule of the β-form is similar to δ-(1,6-DAP)(BA); overlapping with the amino group, and this nonorthogonality in turn affords hole-dominant transport according the to partition theory (Table S5f).

It has been described that nucleation of γ-(1,6-DAP)(BA) is preferential, but δ-(1,6-DAP)(BA) is thermodynamically more stable.^41^ Since we could not detect XRD peaks, we cannot conclude even the thin-film homogeneity of (1,6-DAP)(BA). If the γ-phase is perfectly orthogonal, like α-(1,6-DAP)(CA), purely n-channel transport is expected. However, the 1,6-DAP molecules in one of the four stacks are rotationally disordered^41^ and increase the hole transport. This is reminiscent of the low-temperature rotation in (anthracene)(TCNQ).^62^ We could not exclude the possibility that δ-(1,6-DAP)(BA) affords electron-dominant transport because (DBTTF)(TCNQ) is categorized to hole-dominant ambipolar transport from the viewpoint of the partition theory,^22^ but exhibits mainly n-channel transport depending on the conditions.^63−66^ As another possibility, however, the observed n-channel transport is understandable if the thin film is assumed to be mainly composed of the γ-phase.

In the case of (1,5-DAN)(DMTCNQ), the long axis of DMTCNQ is largely tilted (65°) from the D long axis, but the transfer between the D HOMO and the A LUMO, *t*_DA_, is comparatively small (Table 2). This complex is accidentally orthogonal; as a result, *t*_e_^eff^ is significantly larger than *t*_h_^eff^. This calculation is in good agreement with the observed n-channel transport. (1,5-DAN)(TCNQ) is not obviously orthogonal, but due to the cooperative enhancement from HOMO-2 (Table S3g), *t*_e_^eff^ is again larger than *t*_h_^eff^. (2,3-DAN)(DMTCNQ) is also electron-dominant because *t*_h_^eff^ emerges only from the HOMO/LUMO interaction, whereas *t*_e_^eff^ is enhanced by the contribution of D HOMO-1 since this orbital has the same gerade symmetry as D HOMO. Orbital overlap in (1-MAP)(TCNQ) is clearly orthogonal as the D molecule is positioned exactly on the top of the A molecule.^43^ A remarkably similar overlap has been observed in the newly fabricated (1-MAP)(DMTCNQ) complex (Figure 2m); although we have not observed the transistor properties, electron-dominant transport is predicted due to a similar molecular arrangement. In general, the TCNQ and DMTCNQ complexes are electron-dominant when the long axis of the TCNQ molecule with vertical nodes is located exactly on the top of the D molecule. However, despite any molecular rotation, these complexes exhibit electron-dominant transport due to cooperative enhancement from HOMO-1.

### 4.2 Interplay between Energy Levels and Effective Transfers

When the electrodes of (1,5-DAN)(BA) transistors are replaced by silver, the characteristics are changed from the p-channel to ambipolar (Figure 4b). Transistor characteristics of mixed-stack complexes are influenced not only by the effective transfers but also by the injection barriers.^30,67^

According to the Marcus theory, we can calculate mobility μ^calc^,^68^ to which the transistor current is proportional (Table 3). The mobility of mixed-stack materials is basically proportional to the square of the effective transfer integrals obtained from Table 2. Reorganization energies of the acceptor and donor molecules are used for electron and hole transport, respectively (Table S7).

**Table 3:** Current Ratios Expected from Effective Transfers and Injection Barriers

complex^a^	(*t*_e_^eff^/*t*_h_^eff^)^2^	λ_e_/λ_h_ (meV)	μ_e_^calc^/μ_h_^calc^	*E*_L_ – Φ_M_/Φ_M_ – *E*_H_ (eV)	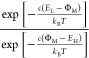	*I*_e_/*I*_h_ (obs)
(1,5-DAN)(FA) C	0.002	676/541	0.00056	0.31/0.22	0.78	0.0004 (10^–4^)
(1,5-DAN)(CA) C	0.052	524/541	0.061	0.43/0.22	0.55	0.033 (10^–4^)
(1,5-DAN)(BA) C silver	0.043	483/541	0.075	0.51/0.22	0.45	0.034 (10^–4^)
				0.03/0.76	9.0	0.67 (0.26)
δ-(1,6-DAP)(BA) Q	0.11	483/551	0.23	0.41/0.0	0.32	0.074 (12)
α-(1,6-DAP)(CA) Q	51^b^	524/551	69^b^	0.33/0.0	0.40	27
β-(1,6-DAP)(CA) Q	0.064	524/551	0.088	0.33/0.0	0.40	0.035
(1,5-DAN)(TCNQ) C	13.3	258/541	30	0.19/0.22	1.09	33 (12)
(1,5-DAN)(DMTCNQ) C	100	286/541	167	0.30/0.22	0.80	133 (25)
(2,3-DAN)(DMTCNQ) C	8.2^b^	286/266	7.4^b^	0.30/0.42	1.5	11 (8)
(1-MAP)(TCNQ) Q	289	258/365	974	0.21/0.09	0.66	642 (2)

a C: carbon, and Q: (TTF)(TCNQ) [−4.7 eV] electrodes.

b From the triad method.

Injection barrier is represented by exp(− *c*(Φ_M_ – *E*_H_)/*k*_B_*T*), where the numerator, Φ_M_ – *E*_H_, coming from the D HOMO level *E*_H_ and the electrode level Φ_M_ is replaced by *E*_L_ – Φ_M_ for electrons (Figure 1). Combining these factors, the ratio of electron and hole currents is represented by the following formula
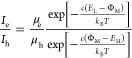
2The actual Schottky barrier Φ_B_ is smaller than the pristine energy-level difference Φ_M_ – *E*_H_ by a factor of *c*. On the basis of electrode dependence of organic transistors (Table S7),^63,69^ we adopt *c* = 0.07 (Supporting Information). Experimental results suggest that less than one-tenth of the electrode work function change is reflected in the Schottky barrier.^70^ By using *E*_H_, *E*_L_, and Φ_M_ taken from Figure 1 together with the effective transfer integrals obtained by partition theory (Table 2), the current ratios are estimated as shown in Table 3. Factors larger than 1 lead to electron-dominant transport, whereas factors smaller than 1 mean hole-dominant transport.

In (1,5-DAN)(BA), due to the large *t*_h_^eff^, the hole transport overwhelms the electron transport and *I*_e_/*I*_h_ is as small as 0.034. However, when the carbon is substituted by silver, the exponential factor increases from 0.45 to 9.0. As a result, electron injection becomes preferable, and *I*_e_/*I*_h_ increases to 0.67, in agreement with the observed hole-dominant ambipolar transport.

δ-(1,6-DAP)(BA) is nonorthogonal, but the same mechanism as (1,5-DAN)(BA) makes this phase hole-dominant (*I*_e_/*I*_h_ ∼ 0.074). However, if the thin-film phase has a stacking structure similar to those of γ-(1,6-DAP)(BA) and α-(1,6-DAP)(CA), the D HOMO and the A LUMO are orthogonal, and n-channel transport is expected. This argument is in agreement with the observed electron-dominant ambipolar transport. Although 1,6-DAP is a much stronger donor than 1,5-DAN, the orbital orthogonality makes (1,6-DAP)(BA) n-channel. In (1,5-DAN)(TCNQ), (1,5-DAN)(DMTCNQ), (2,3-DAN)(DMTCNQ), and (1-MAP)(TCNQ), the large *t*_e_^eff^ leads to n-channel transport (*I*_e_/*I*_h_ > 1). TCNQ has a smaller reorganization energy λ (258 meV) than other donors and acceptors presented here (>500 meV), and this is another reason for n-channel transport observed in these TCNQ complexes.

When the energy level of a donor is situated near the hole transport limit (−5.6 eV), the exponential factor becomes 0.07. From this viewpoint, the observed current is less than one-tenth, and it is reasonable that the hole transport is practically lost.^45^ Taking into account the effective transfers, reorganization energies, and injection barriers, this estimation explains the observed transistor properties sufficiently.

There are several examples in organic transistors in which the charge carrier polarity depends on the electrode materials.^30,64,67^ The present method seems widely applicable to inorganic metal electrodes (Table S8), though it is desirable to extend the present method to include organic metal electrodes.^63^

## 5 Conclusions

Mixed-stack complexes of DAN and DAP have exhibited p-channel, n-channel, and ambipolar transistor properties. Since DAN and DAP are sufficiently strong donors, the carrier charge polarity does not solely depend on the donor/acceptor redox potentials but rather depends on the orbital overlaps. When the D HOMO and A LUMO are not orthogonal, ambipolar transport is expected; however, charge carrier asymmetry is observed due to contribution from the next HOMO orbitals.

**Figure 7:**
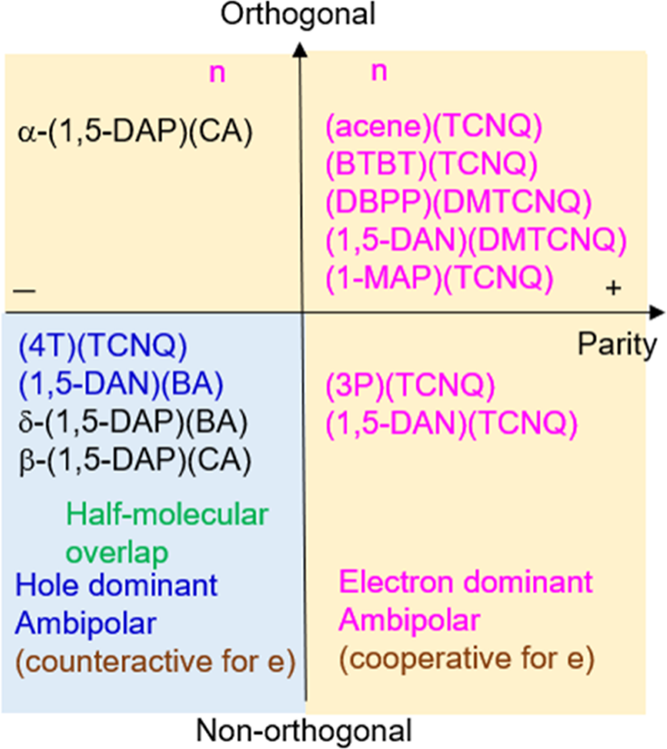
Charge polarity category of charge-transfer complexes. Orthogonality is between the A LUMO and the D HOMO. Parity is between the HOMO and the next important donor orbital. Electron and hole transports have actually been observed in pink and blue materials, respectively. DBPP: dibenzopyrrolopyrrole; 3P: terphenyl.

When the acceptor molecule overlaps with half of the donor molecule, the D HOMO contribution is canceled by the opposite parity of the D HOMO-1 contribution, and the complex shows p-channel transport (Figure 7, bottom left). This cancellation promotes hole transport in (1,5-DAN)(FA), (1,5-DAN)(CA), and (1,5-DAN)(BA). However, using silver electrodes, (1,5-DAN)(BA) still shows hole-dominant ambipolar transport. The competition between transfers and the Schottky barrier is estimated, where the influence of the energy-level difference is reduced by a factor of *c* = 0.07. The previously observed hole-dominant ambipolar transport in (4T)(TCNQ) is understood in the same mechanism.

When the next HOMO orbitals have the same parity as the D HOMO, ambipolar transport is again disturbed. In (1,5-DAN)(TCNQ) and (2,3-DAN)(DMTCNQ), due to the cooperation of HOMO-1 or HOMO-2, the electron transport is larger than the hole transport.

When the acceptor is located exactly on the top of the donor molecule, the D HOMO and A LUMO are orthogonal, and the complex shows mainly n-channel properties (Figure 7, top right). This behavior is observed in (1,5-DAN)(DMTCNQ) and (1-MAP)(DMTCNQ). Although the structure of γ-(1,6-DAP)(BA) is not known, analogy with α-(1,6-DAP)(CA) indicates that the n-channel transport in (1,6-DAP)(BA) is explained along this line. Figure 7 demonstrates that either orthogonality (upper half) or positive parity (right half) makes the material n-channel; thus, TCNQ and DMTCNQ complexes generally show electron-dominant transport. A comparatively small reorganization energy of TCNQ is another reason for the universally observed n-channel transport. Hole-only transport is realized only in the left bottom; orbitals are nonorthogonal, but the second orbital is canceling. Naively speaking, such dominance of electron transport occurs because acceptors are usually smaller molecules than donors, and not capable of mediating hole transport.

For the half-molecule overlap, the partition method affords *t*_e_^eff^ < *t*_h_^eff^, but the triad method gives *t*_e_^eff^ ∼ *t*_h_^eff^. In the triad method, the orbitals are largely reorganized. However, for other cases, the triad and partition methods afford essentially the same results.

Overall, we can predict the carrier charge polarity of mixed-stack complexes from their crystal structures and orbital overlaps. For quinoidal acceptors such as TCNQ and BA, when the DA molecules are regularly stacked only with the molecular long-axis displacement, the complex shows n-channel characteristics. When half-molecule overlap is achieved, p-channel transport is observed. Ambipolar transport is realized in the case of low-symmetry molecular overlaps and when the donor is an oligomer.^45^

Transistor properties of highly conducting segregated complexes are observed in (1,6-DAP)(TCNQ) and (1,6-DAP)(DMTCNQ) by using evaporated thin films. We need not subtract the bulk current using thin films evaporated on bottom-contact (TTF)(TCNQ) electrodes. The characteristics are balanced ambipolar, indicating electrons and holes are equally contributing to the conduction in these practically ionic complexes. This is probably inherent in the Mott insulating state.

## Supporting Information Available

Cyclic voltammetry, crystallographic data, charge-transfer degree, additional transistor characteristics, thin-film properties, transfer integrals of segregated complex, calculation of superexchange transfer integrals, interstack transfer integrals, energy-level diagram of DAN, and estimation of Schottky barriers (PDF)
Crystal data (ZIP)

## Accession Codes

CCDC 2266516–2266522 contain the supplementary crystallographic data for this paper.
