# Supplementary material for: Charge Polarity Control in Organic Transistors of Mixed and Segregated Complexes Based on Diaminonaphthalene and Pyrene
Source: ACS Appl Mater Interfaces. 2023 Sep 15;15(38):45201–11. doi: 10.1021/acsami.3c10583 (PMC10540136; doi:10.1021/acsami.3c10583)
Supplement: Supplementary file 1 — am3c10583_si_001.pdf [file am3c10583_si_001.pdf]

Supporting Information

Charge polarity control in organic transistors of mixed and segregated complexes based on diamino-naphthalene and pyrene

Nikhil Rao Mallela,[†] Tadashi Kawamoto*,[†] and Takehiko Mori*,[†]

Department of Materials Science and Engineering, Tokyo Institute of Technology,
O-okayama, Meguro-ku, Tokyo 152-8552

Cyclic voltammetry

From the redox potentials, the HOMO and LUMO levels were estimated as shown in Table 2.[36]

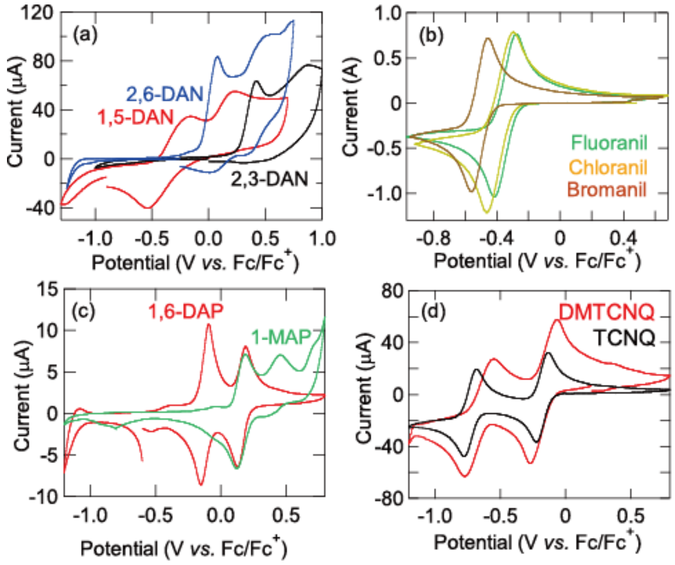

Figure S1. Cyclic voltammograms of (a) DAN, (b) *p*-quinones, (c) DAP, and (d) TCNQ derivatives.

Crystallographic data

Table S1. Crystallographic data of *p*-quinone complexes of 1,5-DAN.

	(1,5-DAN)(FA)[44]	(1,5-DAN)(FA)	(1,5-DAN)(CA)[44]	(1,5-DAN)(BA)[44]	(1,5-DAN)(BA)
Formula	$C_{16}H_{10}F_4N_2O_2$	$C_{16}H_{10}F_4N_2O_2$	$C_{16}H_{10}Cl_4N_2O_2$	$C_{16}H_{10}Br_4N_2O_2$	$C_{16}H_{10}Br_4N_2O_2$
Formula weight	338.26	338.26	404.06	581.90	581.88
Crystal System	Monoclinic	Monoclinic	Monoclinic	Monoclinic	Monoclinic
Space Group	$P2_1/c$	$P2_1/c$	Pn	$P2_1/n$	$P2_1/n$
Shape	Needle	Needle	Needle	Needle	Needle
a (Å)	7.7427(10)	7.8213(19)	6.53070(10)	6.73710(10)	8.047(3)
b (Å)	6.4919(10)	6.5452(15)	7.84010(10)	15.4659(3)	15.556(4)
c (Å)	12.8637(9)	13.034(5)	15.0342(11)	7.9345(6)	6.815(3)
α (deg.)	90	90	90	90	90
β (deg.)	91.185(6)	91.99(3)	98.552(7)	94.480(7)	95.33(3)
γ (deg.)	90	90	90	90	90
V (Å^3)	646.45(5)	666.8(3)	761.21(6)	824.21(7)	849.5(5)
Z-value	2	2	2	2	2
D_{calc} (g cm^{-3})					
Total reflns.		2301			2789
Unique reflns. (R_{int})		1943			1578
$R_1[F^2 > 2\sigma(F^2)]$		0.0510			0.0708
wR_2[All reflections]		0.1593			0.2056
GOF		1.002			1.003
T (K)	85	296	85	85	296

Table S2. Crystallographic data of 1,5-DAN and 2,3-DAN Complexes.

	(1,5-DAN) (TCNQ)	(1,5-DAN) (DMTCNQ)	(2,3-DAN) (TCNQ)	(2,3-DAN) (DMTCNQ)	(1-MAP) (DMTCNQ)
Formula	$C_{22}H_{14}N_8$	$C_{24}H_{18}N_6$	$C_{22}H_{14}N_6$	$C_{24}H_{18}N_6$	$C_{30}H_{19}N_5$
Formula weight	362.39	390.45	362.39	390.45	449.51
Crystal System	Triclinic	Monoclinic	Triclinic	Triclinic	Triclinic
Space Group	$P\bar{1}$	$P2_1/c$	$P\bar{1}$	$P\bar{1}$	$P\bar{1}$
Shape	Needle	Needle	Needle	Needle	Needle
a (Å)	6.9074(3)	7.909(3)	6.654(2)	8.886(8)	6.8444(2)
b (Å)	8.0322(3)	9.188(6)	11.038(3)	9.274(9)	8.7778(5)
c (Å)	8.9758(4)	13.523(6)	13.204(4)	13.192(3)	10.4827(4)
α (deg.)	67.792(3)	90	90.58(2)	105.26(4)	106.625(2)
β (deg.)	69.782(3)	96.99(3)	94.34(2)	100.57(4)	101.736(2)
γ (deg.)	75.999(2)	90	96.83(2)	101.34(7)	102.623(2)
V (Å^3)	429.07(3)	975.4(9)	960.1(4)	996.1(14)	564.55(5)
Z-value	1	2	2	2	1
D_{calc} (g cm^{-3})	1.402	1.329	1.309	1.302	1.322
Total refls.	7423	2729	4519	5101	9246
Unique refls. (R_{int})	1541(0.0796)	2239(0.0326)	3558(0.0428)	4130(0.1604)	1800 (0.0735)
R_1 [$F^2 > 2\sigma(F^2)$]	0.0618	0.0648	0.0514	0.0585	0.0995
wR_2 [All refls.]	0.1759	0.1674	0.1249	0.1487	0.2477
GOF	1.097	1.015	0.959	0.922	0.825
T (K)	106	297	297	298	293

Charge-transfer degree

The charge transfer degree ρ of the TCNQ complexes were estimated from the bond lengths $b \sim d$ and the C≡N stretching mode $\nu_{C\equiv N}$ of the IR spectra by using the following formula.[S1-S10]

$$\rho_{BL} = -41.667\ [c/(b + d)] + 19.818$$

$$\nu_{C\equiv N} = 2227 - 44\rho_{IR}$$

Table S3. Charge-transfer degree

Acceptor	TCNQ			DMTCNQ		
Donor	1,5-DAN	2,6-DAN	2,3-DAN	1,5-DAN	2,6-DAN	2,3-DAN
b (Å)	1.446	—	1.443	1.4515	—	1.4525
c (Å)	1.38	—	1.367	1.374	—	1.3695
d (Å)	1.435	—	1.438	1.436	—	1.4362
ρ_{BL}	0.06	—	0.11	0.00	—	0.01
$\nu_{C\equiv N}$ (cm^{-1})	2213	2210	2216	2210	2207	2212
ρ_{IR}	0.31	0.38	0.25	0.38	0.45	0.33

Acceptor<br>Donor	(1-MAP)<br>(TCNQ)	(1-MAP)<br>(DMTCNQ)	(1,6-DAP)<br>(TCNQ)	(1,6-DAP)<br>(DMTCNQ)
b (Å)	1.437	1.453	1.428	—
c (Å)	1.380	1.390	1.417	—
d (Å)	1.426	1.429	1.418	—
ρ_{BL}	0.25 ± 0.02	0.23 ± 0.05	1.06 ± 0.00	—
$\nu_{C\equiv N}$ (cm^{-1})	2217	2211	2184	2180
ρ_{IR}	0.23	0.36	0.98	1.06

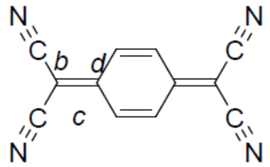

Charge-transfer absorption

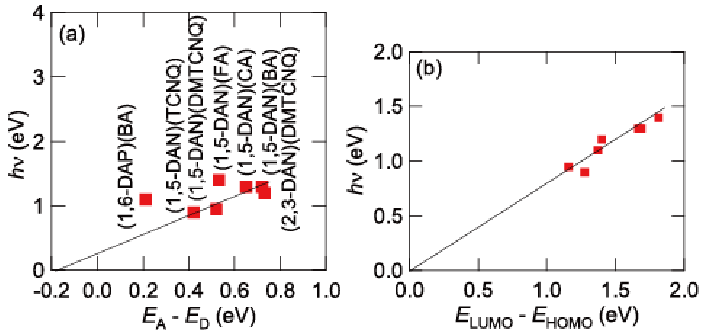

Figure S2. Observed charge-transfer transition plotted against (a) the difference of the individual donor and acceptor levels (Figure 1), and (b) the D HOMO and A LUMO difference calculated from the dimer (Table S5).

Transistor properties

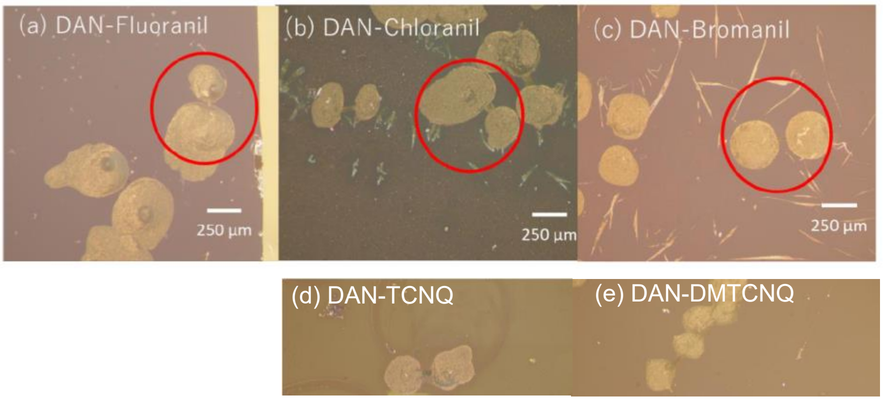

Figure S3. Single-crystal transistors of (a) (1,5-DAN)(FA), (b) (1,5-DAN)(CA), (c) (1,5-DAN) (BA) (d) (1,5-DAN)(TCNQ), and (e) (1,5-DAN)(DMTCNQ) (the same scale).

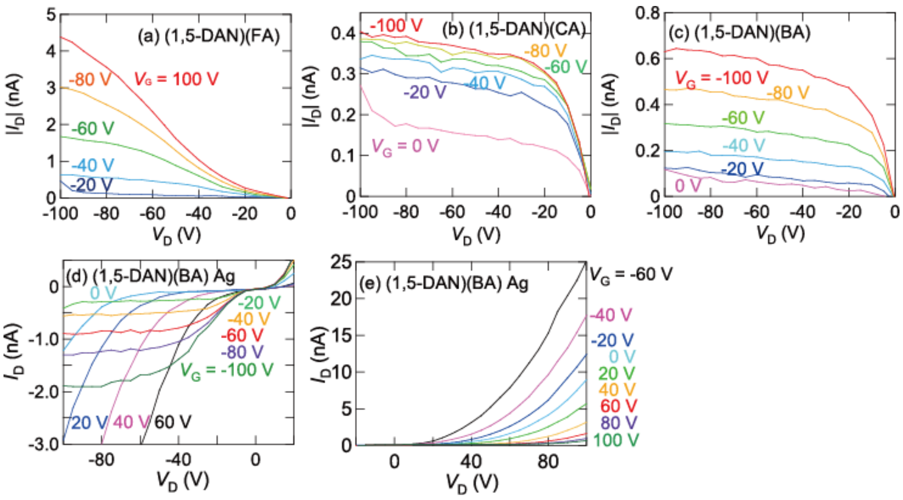

Figure S4. Output characteristics of single-crystal transistors of (a) (1,5-DAN)(FA), (b) (1,5-DAN)(CA), (c) (1,5-DAN)(BA) for carbon electrodes, and (d,e) (1,5-DAN)(BA) for Ag electrodes.

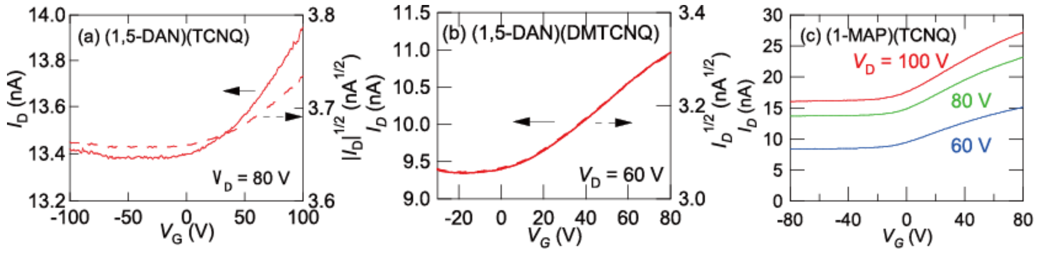

Figure S5. Transfer characteristics of single-crystal transistors of (a) (1,5-DAN)(TCNQ), and (b) (1,5-DAN)(DMTCNQ). (c) Transfer characteristics of a thin-film transistor of (1-MAP)(TCNQ).

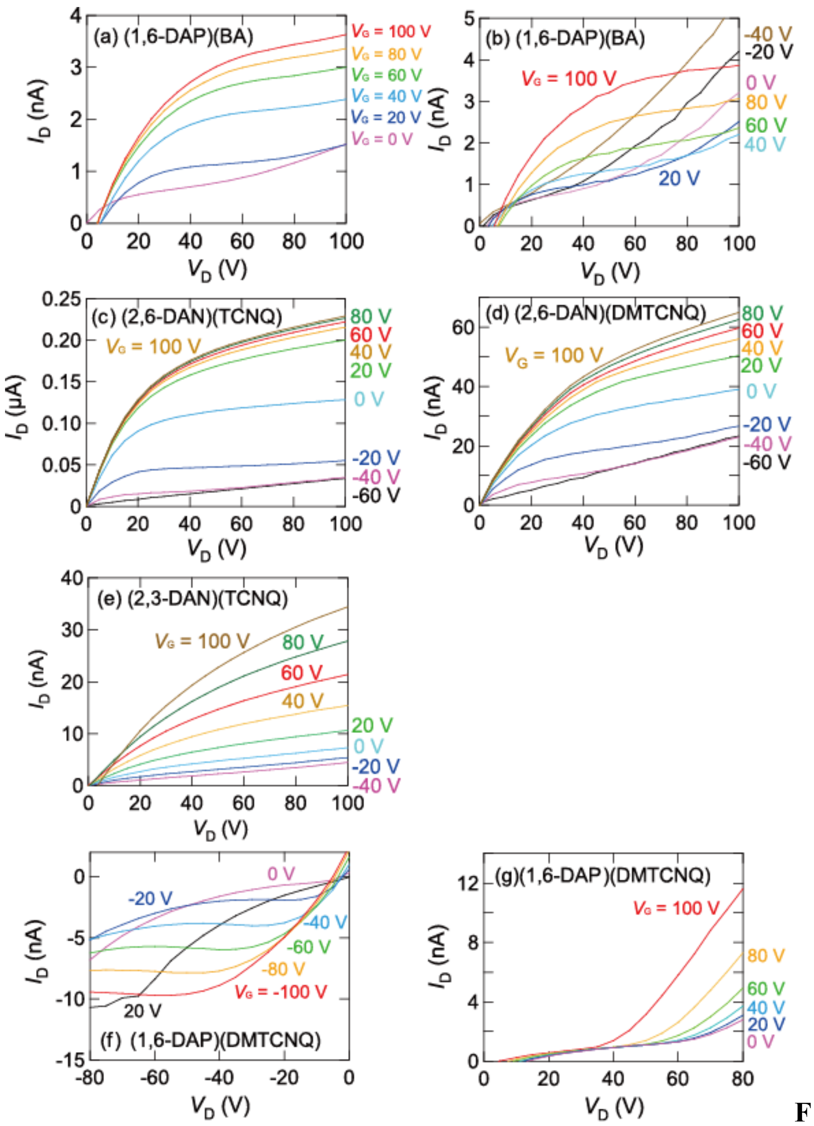

F

Figure S6. Output characteristics of thin-film transistors of (a,b) (1,6-DAP)(BA) (different samples), (c) (2,6-DAN)(TCNQ), and (d) (2,6-DAN)(DMTCNQ). (e) Output characteristics of a single-crystal transistor of (2,3-DAN)(TCNQ). (f,g) Output characteristics of a thin-film transistor of (1,6-DAN)(DMTCNQ).

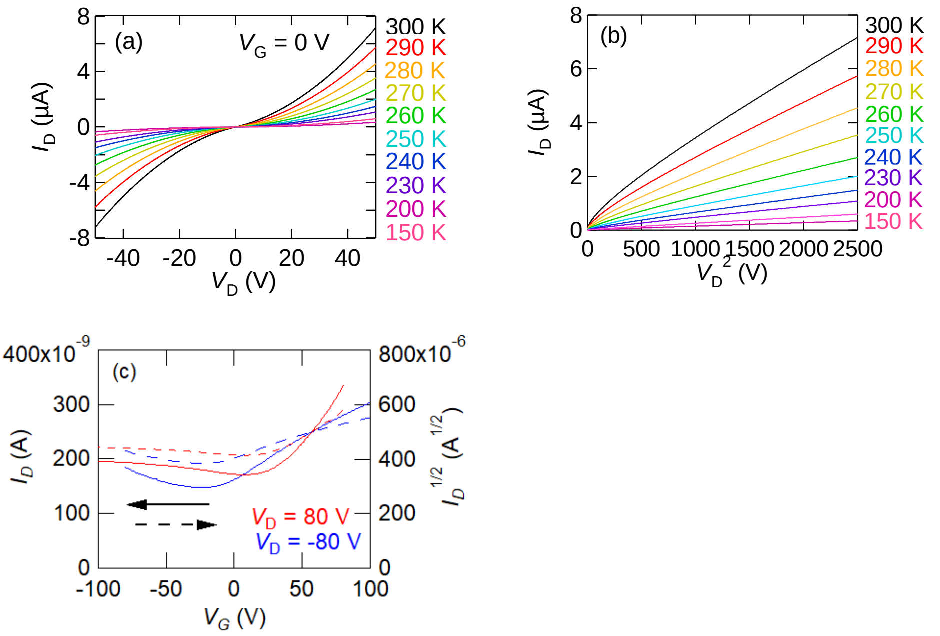

Figure S7. (a) V_D dependence of I_D in a thin film of (1,6-DAP)(TCNQ), and (b) the V_D^2 plot. (c) Transfer characteristics of a thin-film (35 nm on Cytop) transistor of (1,6-DAP)(TCNQ).

Thin-film properties

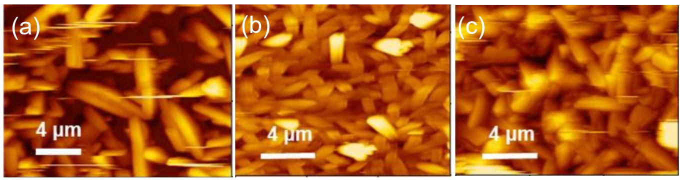

Figure S8. AFM images of (a) (1,6-DAP)(TCNQ), and (b) (1,6-DAP)(DMTCNQ) thin films (35 nm) evaporated on PS. AFM image of (c) (1,6-DAP)(TCNQ) thin films (35 nm) evaporated on OTMS.

X-ray diffraction (XRD) patterns of evaporated films of (1,6-DAP)(TCNQ) and (1,6-DAP)(DMTCNQ) are shown in Figure S9. The peak at d = 6.8 Å in (1,6-DAP)(TCNQ) corresponds to the side-on arrangement (~6.5 Å),[S11] rather than the tail-on arrangement (9-10 Å). Since we could not determine the crystal structure of (1,6-DAP)(DMTCNQ), we cannot assign the layer spacing of d = 8.97 Å.

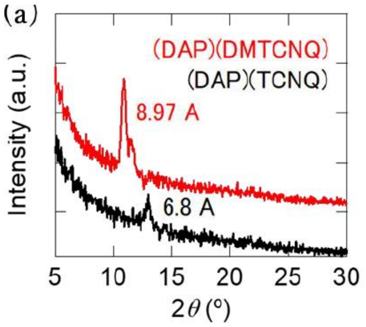

Figure S9. X-ray diffraction of (1,6-DAP)(TCNQ), and (1,6-DAP)(DMTCNQ) evaporated on OTMS.

Table S4. Calculated transfer integrals of (1,6-DAP)(TCNQ).

Stack			Interstack			
t_d	t_a	t_q	t_p	t_l	t_m	t_n
188	213	59	61	59	61	75

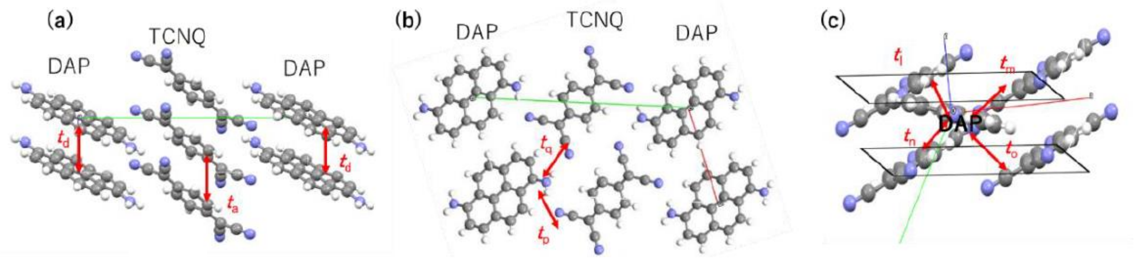

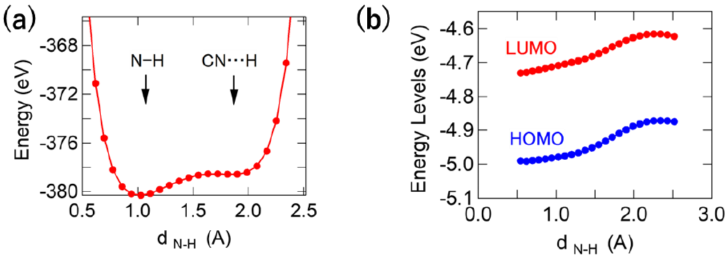

Figure S10. Calculated (a) intermolecular energy, and (b) energy level shift for proton transfer in (1,6-DAP)(TCNQ).[54]

Superexchange transfer integrals

Table S5. Energy difference, transfers, and contributions to superexchange transfers (meV) in the partition calculation.

(a) (1,5-DAN)(FA)

Electron				Hole			
$A_{LUMO}\rightarrow$	$E_i - E_0$	t	$t^2/(E_i - E_0)$	$D_{HOMO}\rightarrow$	$E_i - E_0$	t	$t^2/(E_i - E_0)$
D_{LUMO}	–2378	75	2	A_{LUMO+1}	–5171	76	-1
D_{HOMO}	1815	341	64	A_{LUMO}	–1815	341	**64**
D_{HOMO-1}	2993	445	-66	A_{HOMO}	2256	150	-10
D_{HOMO-2}	3352	19	0	A_{HOMO-1}	2562	156	9
D_{HOMO-3}	5170	125	3	A_{HOMO-2}	2590	5	0
Total			**3**	Total			**62**

(b) (1,5-DAN)(CA)

Electron				Hole			
$A_{LUMO}\rightarrow$	$E_i - E_0$	t	$t^2/(E_i - E_0)$	$D_{HOMO}\rightarrow$	$E_i - E_0$	t	$t^2/(E_i - E_0)$
D_{LUMO}	–2591	-86	3	A_{LUMO+1}	–4158	72	–1
D_{HOMO}	1687	-300	**53**	A_{LUMO}	–1687	300	**53**
D_{HOMO-1}	2793	356	**–45**	A_{HOMO}	1866	34	–1
D_{HOMO-2}	3201	39	0	A_{HOMO-1}	2347	116	6
D_{HOMO-3}	4940	177	1	A_{HOMO-2}	2367	27	0
Total			**13**	Total			**57**

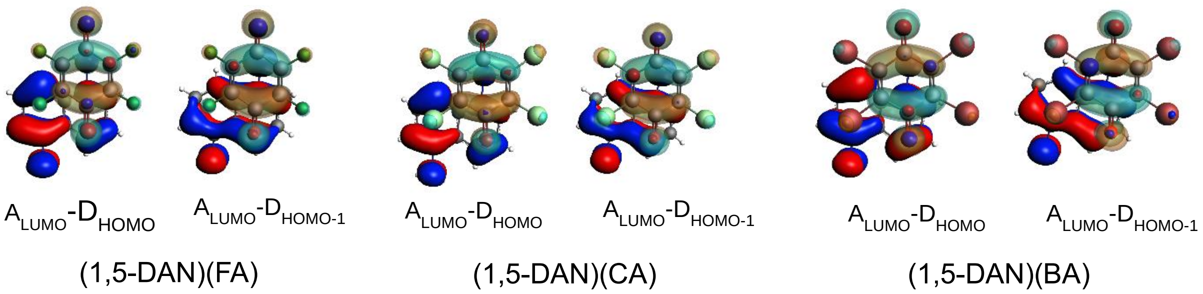

(c) (1,5-DAN)(BA)

Electron				Hole			
A_{LUMO}→	$E_i - E_0$	t	$t^2/(E_i - E_0)$	D_{HOMO}→	$E_i - E_0$	t	$t^2/(E_i - E_0)$
D_{LUMO}	–2321	-81	3	A_{LUMO+1}	–3662	63	–1
D_{HOMO}	1400	278	**55**	A_{LUMO}	–1400	278	**55**
D_{HOMO-1}	2502	362	**–52**	A_{HOMO}	1677	31	–1
D_{HOMO-2}	2878	56	1	A_{HOMO-1}	1812	88	4
D_{HOMO-3}	4570	159	6	A_{HOMO-2}	2111	12	0
Total			**12**	Total			**58**

(d) δ-(1,6-DAP)(BA)

Electron					Hole				
A_{LUMO}	$E_i - E_0$	t_1	t_2	$t_1t_2/(E_i - E_0)$	D_{HOMO}→	$E_i - E_0$	t_1	t_2	$t_1t_2/(E_i - E_0)$
D_{LUMO}	–2082	84	–70	3	A_{LUMO+1}	–3782	1	6	0
D_{HOMO}	1372	195	178	**25**	A_{LUMO}	–1372	195	178	**25**
D_{HOMO-1}	2768	227	–13	–1	A_{HOMO}	1954	–74	–52	–2
D_{HOMO-2}	2859	224	–257	**–20**	A_{HOMO-1}	2200	–134	104	6
D_{HOMO-4}	4688	211	–70	3					
Total				**10**	Total				**30**

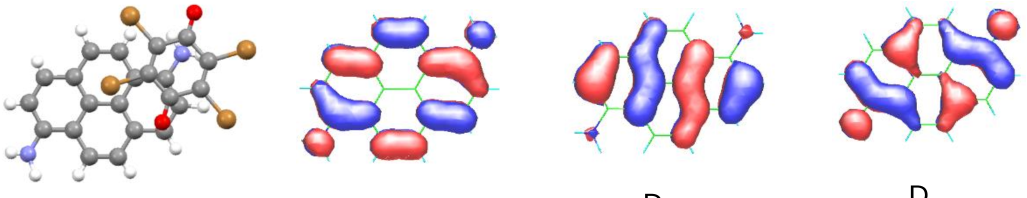

δ-(1,6-DAP)(BA) D_{HOMO} D_{HOMO-1} D_{HOMO-2}

When the amino group is regarded as the "para" position of the side ring, the HOMO and HOMO-2 have the same symmetry at the side ring. Then, the contribution of HOMO-2 cancels the HOMO interaction analogously to (1,5-DAN)(BA), and this phase becomes hole dominant. The same mechanism applies to β-(1,6-DAP)(CA) as well (Table S5(f)). Although the crystal structure of δ-(1,6-DAP)(BA) has been solved using a space group *Pn*,[41] the structure seems centrosymmetric. After moving the DAP molecule to the origin and averaging, the calculation has given essentially the same result: 3/28 meV.

(e) α-(1,6-DAP)(CA)

Electron				Hole			
A_{LUMO}→	$E_i - E_0$	t	$t^2/(E_i - E_0)$	D_{HOMO}→	$E_i - E_0$	t	$t^2/(E_i - E_0)$
D_{HOMO}	933	23	1	A_{LUMO}	–933	23	1
D_{HOMO-1}	2403	185	**14**	A_{HOMO}	2598	66	–2
D_{HOMO-2}	2478	119	–5	A_{HOMO-1}	3238	21	0
D_{HOMO-3}	3192	25	0				0
Total			**9**	Total			**1**

(f) β-(1,6-DAP)(CA)

Electron				Hole			
A_{LUMO}→	$E_i - E_0$	t	$t^2/(E_i - E_0)$	D_{HOMO}→	$E_i - E_0$	t	$t^2/(E_i - E_0)$
D_{HOMO}	933	230	**57**	A_{LUMO}	–933	230	**57**
D_{HOMO-1}	2403	78	3	A_{HOMO}	2598	10	0
D_{HOMO-2}	2478	332	**–44**	A_{HOMO-1}	3238	144	6
D_{HOMO-3}	3192	35	0				
Total			**16**	Total			**63**

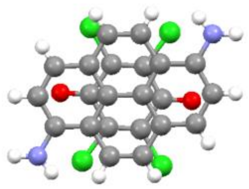

α-(1,6-DAP)(CA)

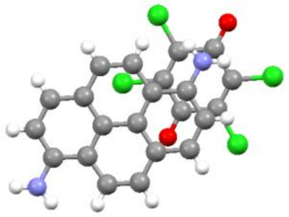

β-(1,6-DAP)(CA)

(g) (1,5-DAN)(TCNQ)

Electron				Hole			
A_{LUMO}→	$E_i - E_0$	t	$t^2/(E_i - E_0)$	D_{HOMO}→	$E_i - E_0$	t	$t^2/(E_i - E_0)$
D_{LUMO}	–3050	105	4	A_{LUMO+1}	–4109	16	0
D_{HOMO}	1273	161	**20**	A_{LUMO}	–1273	161	**20**
D_{HOMO-1}	2245	83	–3	A_{HOMO}	1412	133	13
D_{HOMO-2}	2945	375	**48**	A_{HOMO-1}	2792	136	–7
D_{HOMO-3}	4355	140	5	A_{HOMO-2}	3602	157	–7
Total			**73**	Total			**20**

(1,5-DAN)(TCNQ)

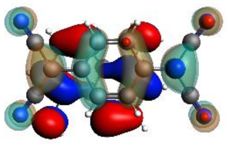

A_{LUMO}-D_{HOMO}

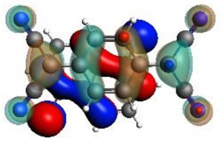

A_{LUMO}-D_{HOMO-1}

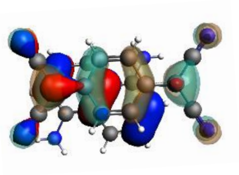

A_{LUMO}-D_{HOMO-2}

(h) (1,5-DAN)(DMTCNQ)

Electron				Hole			
A_{LUMO}→	$E_i - E_0$	t	$t^2/(E_i - E_0)$	D_{HOMO}→	$E_i - E_0$	t	$t^2/(E_i - E_0)$
D_{LUMO}	–3083	32	0	A_{LUMO+1}	–4045	128	4
D_{HOMO}	1159	-101	9	A_{LUMO}	–1159	101	9
D_{HOMO-1}	2173	-226	**23**	A_{HOMO}	1588	29	1
D_{HOMO-2}	2801	-84	3	A_{HOMO-1}	2610	137	–7
D_{HOMO-3}	4445	147	5	A_{HOMO-2}	3762	90	–2
Total			**40**	Total			**4**

(1,5-DAN)(DMTCNQ)

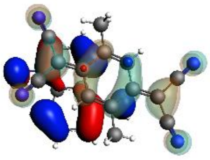

A_{LUMO}-D_{HOMO}

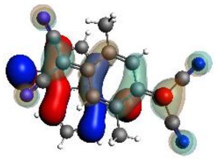

A_{LUMO}-D_{HOMO-1}

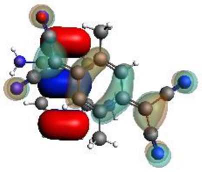

A_{LUMO}-D_{HOMO-2}

(i) (2,3-DAN)(DMTCNQ)

Electron					Hole					
A_{LUMO}	$E_i - E_0$	t_1	t_2	$t_1 t_2/(E_i - E_0)$	D_{HOMO}→	$E_i - E_0$	t_1	t_2	$t^2/(E_i - E_0)$	
D_{LUMO}	-2829	110	52	2	A_{LUMO+1}	–4509	–1	48	0	1
D_{HOMO}	1667	–317	133	**25**	A_{LUMO}	–1667	–317	133	**60**	**11**
D_{HOMO-1}	1791	159	42	–4	A_{HOMO}	1043	–70	96	5	9
D_{HOMO-2}	3465	4	–6	0	A_{HOMO-1}	2167	–64	36	2	–1
D_{HOMO-3}	4062	–95	47	–1	A_{HOMO-2}	3108	–254	7	-22	0
Total				**22**	Total				**45**	**20**

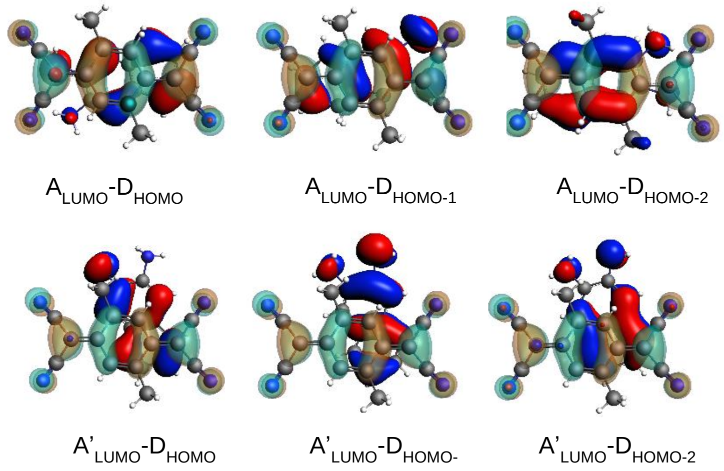

(2,3-DAN)(DMTCNQ)

(j) (1-MAP)(TCNQ)

Electron				Hole			
A_{LUMO}→	$E_i - E_0$	t	$t^2/(E_i - E_0)$	D_{HOMO}→	$E_i - E_0$	t	$t^2/(E_i - E_0)$
D_{LUMO}	–2285	66	–2	A_{LUMO+1}	–3641	33	0
D_{HOMO}	817	90	**10**	A_{LUMO}	–817	90	**–10**
D_{HOMO-1}	2112	353	**59**	A_{HOMO}	1557	79	4
D_{HOMO-2}	2685	63	1	A_{HOMO-1}	3080	11	0
D_{HOMO-3}	2838	6	0	A_{HOMO-2}	3538	78	2
Total			**68**	Total			**–4**

(k) (1-MAP)(DMTCNQ)

Electron				Hole			
A_{LUMO}→	$E_i - E_0$	t	$t^2/(E_i - E_0)$	D_{HOMO}→	$E_i - E_0$	t	$t^2/(E_i - E_0)$
D_{LUMO}	–2387	2	0	A_{LUMO+1}	–3903	33	0
D_{HOMO}	1067	40	**2**	A_{LUMO}	–1067	90	**–2**
D_{HOMO-1}	2365	346	**51**	A_{HOMO}	1563	79	0
D_{HOMO-2}	2982	126	5	A_{HOMO-1}	2714	11	2
D_{HOMO-3}	3223	38	0	A_{HOMO-2}	3064	78	0
Total			**58**	Total			**–1**

Inter-stack transfer integrals

It has been reported that when there are hydrogen bonds, inter-stack transfer integrals are extraordinarily large, which typically amount to one third of the intra-stack transfer integrals.[54] The same is observed when the hydrogen bond exists between the donor and acceptor molecules. The triad method affords considerable inter-stack effective transfer integrals along the hydrogen bonds (Table S6), which sometimes amount to more than half of the intra-stack effective transfer integrals. Hydrogen bonds appear between almost coplanar molecules. It is characteristic that these intra-stack effective transfer integrals are approximately the same for electron and hole. However, the partition method gives practically zero (< 1 meV) effective transfer integrals for the same interactions, and physical meaning of these inter-stack transfer integrals is not certain.

Table S6. Inter-stack effective transfer integrals along the hydrogen bonds (meV).

Complex	N−O (N) (Å)	Triad
		t_e^{eff}/ t_h^{eff}
(1,5-DAN)(FA)	3.06	22/22
(1,5-DAN)(CA)	2.94	25/26
(1,5-DAN)(BA)	3.00	27/28
δ-(1,6-DAP)(BA)	2.92, 3.13	33/35
α-(1,6-DAP)(CA)	3.10	16/16
β-(1,6-DAP)(CA)	3.01	20/21
(1,5-DAN)(TCNQ)	3.22	DD 11
	3.21	15/16
(1,5-DAN)(DMTCNQ)	3.28	9/10
	3.32	9/16

(DAN)(BA) [(DAN)(FA) and (DAN)(CA) have basically the same arrangements.]

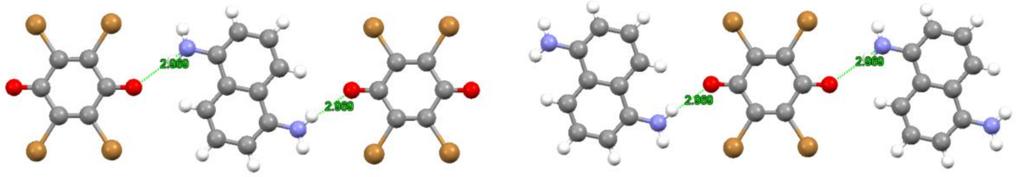

δ-(DAP)(BA)

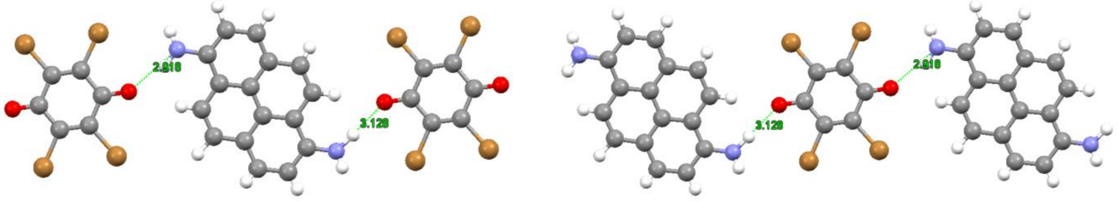

α-(DAP)(CA)

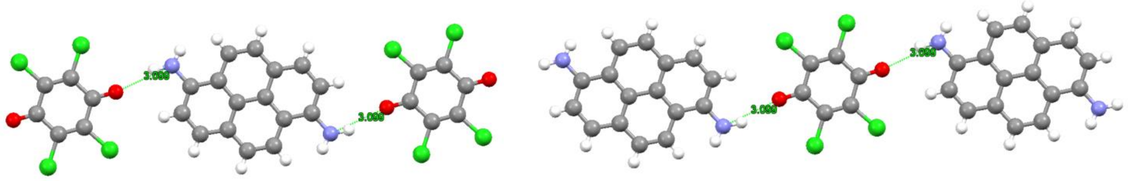

β-(DAP)(CA)

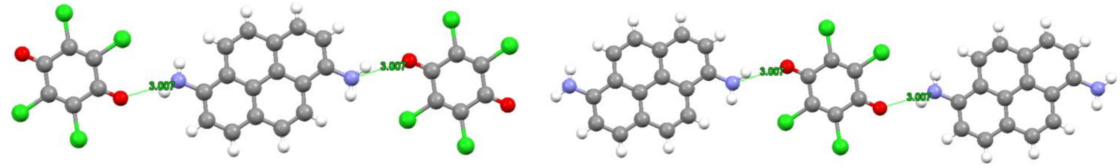

(DAN)(TCNQ)

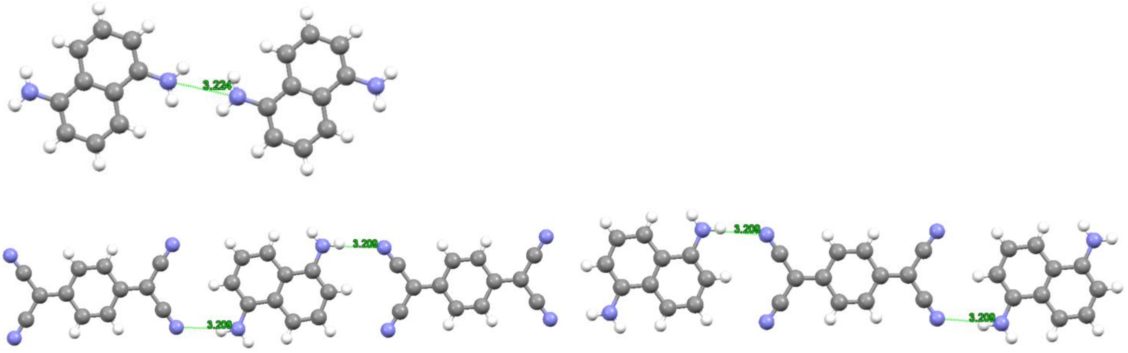

(DAN)(DMTCNQ)

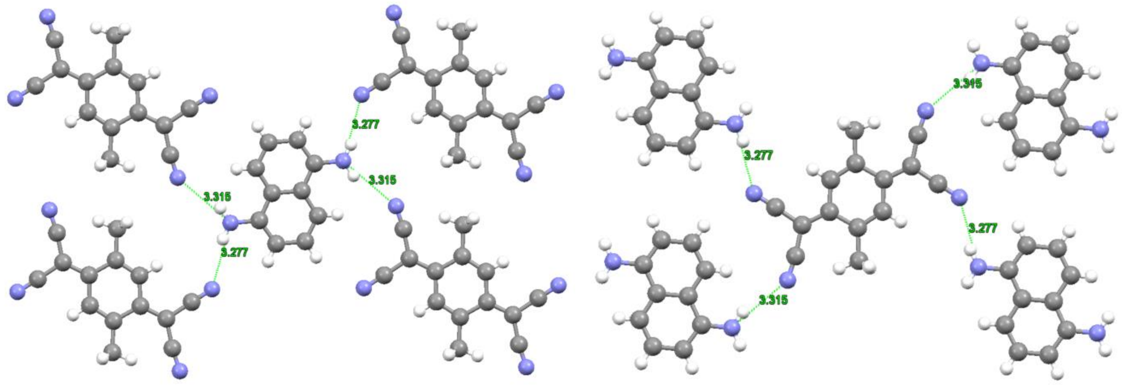

Molecular orbital levels of diaminonaphthalenes

In 1,5-DAN, the HOMO and HOMO-1 come from the antibonding and bonding combinations of the same orbital (Figure 6(b)). Since orbital overlap is important in controlling transistor properties, the molecular orbitals are analyzed. Figure S11(a) demonstrates how molecular orbitals of naphthalene are influenced by successive amino group substitutions at various positions. Naphthalene HOMO and HOMO-2 have a horizontal node, while HOMO-1 has vertical nodes. Addition of an amino group at the 1 (pink) and 2 (green) positions significantly increases the HOMO energy levels. 2-Substitution increases the (green) HOMO-1 energy level, whereas 1-substitution leads to an insignificant increase of the (pink) HOMO-1 level. In general, when the molecular orbital has a large amplitude at the amino substituted carbon, the energy level increases significantly, whereas when the amino carbon corresponds to a node of the molecular orbital, the energy level does not change largely. For diamino substitutions, amino groups at 1 and 8 positions considerably increase the (pink) energy level of HOMO-2 (horizontal node) superseding energy of HOMO-1 (vertical node), resulting in shape exchange between HOMO-2 and HOMO-1. Accordingly, the (pink) HOMO-1 of 1,5-DAN (rightmost in Figure S11(a)) comes from the naphthalene HOMO-2, and has a similar horizontal node to the HOMO. Addition at positions 2 and 3, however, even reduces the (green) HOMO energy of 2,3-DAN as the out-of-plane lone pairs on nitrogen do not contribute to the increase in the energy level, but rather the inductive effect is more significant due to the steric hinderance from the neighboring hydrogen on the nitrogen molecules. 2,6-DAN and 1,5-DAN are moderately strong donors; the HOMO levels go up by 1 eV in comparison with the parent naphthalene, and are situated around -5 eV. By contrast, 2,3-DAN is a much weaker donor with the HOMO level around -5.2 eV.

The same principle applies to pyrene (Figure S11(b)). The 1-substitution (pink) increases the HOMO level, but does not increase HOMO-1. The opposite happens in the 2-substitution (green). As a result, HOMO and HOMO-1 are approximately degenerated in 2-aminopyrene, whereas 1-aminopyrene has a large (1.3 eV) gap between HOMO and HOMO-1. In 1,6-DAP, HOMO-2 is nearly degenerated with HOMO-1. Although this orbital is still HOMO-2, the mechanism of the cancellation is entirely the same as 1,5-DAN (Table S5(c)).

In aminoacenes, energy levels of orbitals with a large amplitude at the amino substituted carbon go up, but at the same time have a large overlap with BA LUMO. As a consequence, it is likely that complexes forming half-molecule overlap suffer from the cancellation of electron transport and show p-channel transport.

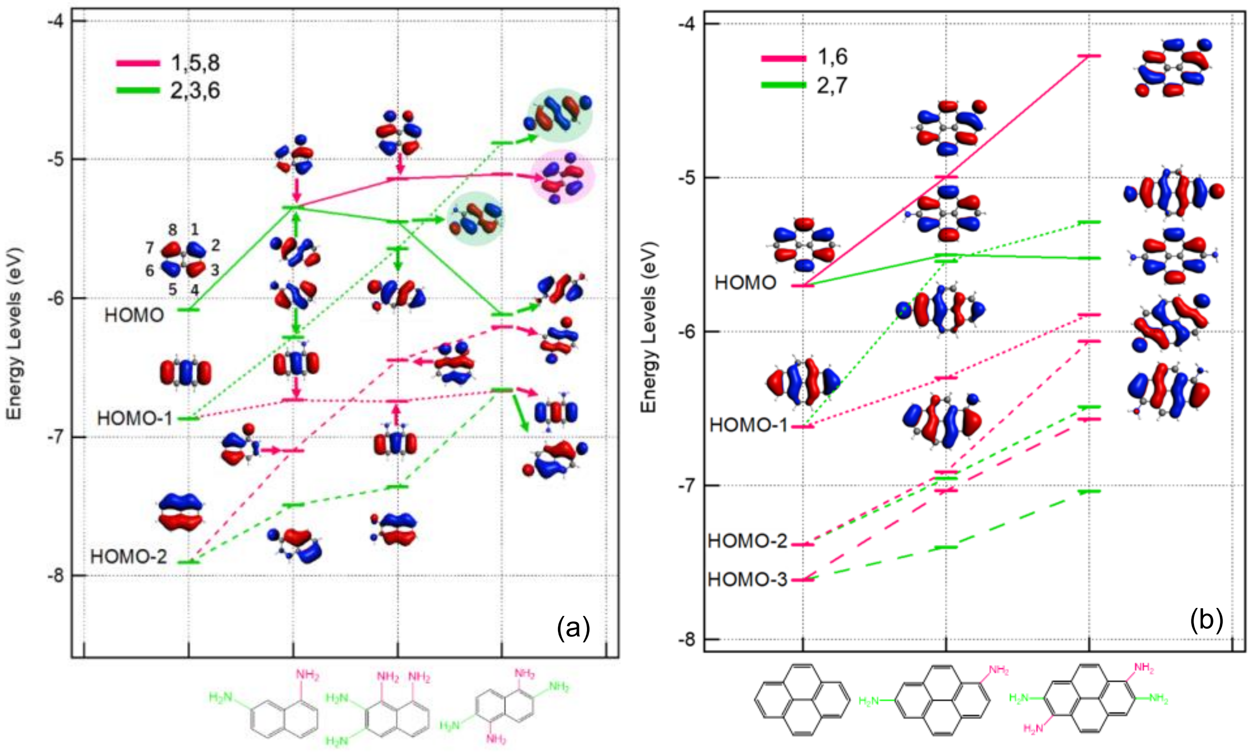

Figure S11. Calculated energy levels and molecular orbitals of (a) amino naphthalene, and (b) amino pyrene.

Calculated mobilities

Mobility is calculated according to the Marcus theory.[68,S12,S13]

$$\mu^{calc} = \frac{1}{2n}\frac{4\pi\sqrt{\pi}ea^2t^2}{hk_BT}F \qquad F = \sqrt{\frac{1}{4\lambda k_BT}}\exp\left(-\frac{\lambda}{4k_BT}\right)$$

The factor including the reorganization energy λ is designated as F.[S14] Table S7 shows the calculated results. If λ is nearly the same, μ is proportional to t square. In general, λ decreases with extending aromatic rings; for example, naphthalene (189) to pentacene (99 meV),[S15] and dithiophene (365) to sexithiophene (253 meV).[S16] Although TCNQ is a small molecule, TCNQ has a small λ (258 meV) compared with other donors and acceptors.[S13,S17] This affords by one order larger F = 0.50 eV^{-1} than F = 0.042 eV^{-1} in BA (λ = 483 meV). Although it is difficult to prepare thin films of TCNQ, a large mobility close to 1 cm^2 V^{-1} s^{-1} has been observed in TCNQ.[S18-S20] Recently, significant isomer dependence of λ has been reported in fused thiophenes.[S21] Accordingly, a small λ has a significant impact to μ.

Table S7. Transfer integral t, reorganization energy λ, and calculated mobility μ

Compound	a (Å)	t (meV)	λ (meV)	F (eV^{-1})	μ^{calc} ($cm^2\ V^{-1}\ s^{-1}$)
(1,5-DAN)(FA)	7.743	e 3	676	0.0054	1.0×10^{-5}
		h 62	541	0.022	0.018
(1,5-DAN)(CA)	6.531	e 13	524	0.027	6.7×10^{-4}
		h 57	541	0.022	0.011
(1,5-DAN)(BA)	6.815	e 12	483	0.042	9.7×10^{-4}
		h 58	541	0.022	0.013
δ-(1,6-DAP)(BA)	9.891	e 10	483	0.042	0.0014
		h 30	551	0.020	0.0061
α-(1,6-DAP)(CA)	6.730	e 43 [a]	524	0.027	0.0079
		h 6 [a]	551	0.020	1.1×10^{-4}
β-(1,6-DAP)(CA)	6.791	e 16	524	0.027	0.0011
		h 63	551	0.020	0.013
(1,5-DAN)(TCNQ)	6.907	e 73	258	0.50	0.45
		h 20	541	0.022	0.015
(1,5-DAN)(DMTCNQ)	7.909	e 40	286	0.36	0.13
		h 4	541	0.022	7.8×10^{-4}
(2,3-DAN)(DMTCNQ)	7.057	e 63 [a]	286	0.36	0.25
		h 22 [a]	266	0.41	0.034
(1-MAP)(TCNQ)	6.786	e 68	258	0.50	0.37
		h 4	365	0.15	3.8×10^{-4}

[a] From the triad results. Others are from the partition results.

Estimation of Schottky barriers

Schottky barrier Φ_B is usually smaller than the pristine energy level difference, $\Phi_M - E_H$.

$$\Phi_B = c(\Phi_M - E_H)$$

For silicon, the actual barrier Φ_B is by c = 0.27 times smaller than the difference of the work functions.[S23] Photoelectron spectroscopy has demonstrated that Φ_B is typically half of the energy level difference in organic semiconductors.[69,70,S23-S26] The c values estimated from electrode material dependence of mobility in organic transistors are listed in Table S8. From this, we have used the average c = 0.07 in Table 3. Such remarkable reduction of Schottky barrier has been attributed to surface states or defects,[S22] and interfacial charge transfer and dipole formation.[70] The figure attached to Table S8 demonstrates that eq 2 is universally applicable to inorganic metals such as Au, Cu, Ag, and Mg, though Ca is somewhat deviated.

Table S8. c values obtained from electrode dependence.

Materials	c	Ref.
Pentacene	0.062	S26
Pentacene	0.123	69
Pentacene	0.060	S27
Perylene	0.070	S28
CuPc	0.086	S26
DBTTF	0.066	63
DMDCNQI	0.070	S29

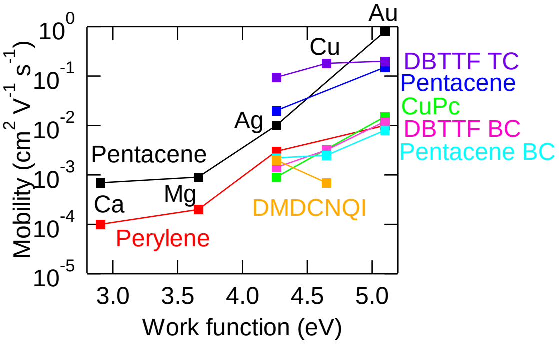

References

(S1) Chappell, J. S.; Bloch, A. N.; Bryden, W. A.; Maxfield, M.; Poehler, T. O.; Cowan, D. O. Degree of Charge Transfer in Organic Conductors by Infrared Absorption Spectroscopy. *J. Am. Chem. Soc.* **1981**, *103*, 2442–2443.

(S2) Wozniak, W. T.; Depasquali, G.; Klein, M. V.; Sweany, R. L.; Brown, T. L. Vibrational Spectra of TTF-TCNQ: Evidence for TTF^0 and $TCNQ^0$ in Thin Films. *Chem. Phys. Lett.* **1975**, *33*, 33–36.

(S3) Khanna, S. K.; Pouget, J. P.; Comès, R.; Garito, A. F.; Heeger, A. J. X-ray Studies of $2k_F$ and $4k_F$ Anomalies in Tetrathiafulvalene-Tetracyanoquinodimethane (TTF-TCNQ). *Phys. Rev. B* **1977**, *16*, 1468–1479.

(S4) Kagoshima, S.; Ishiguro, T.; Anzai, H. X-Ray Scattering Study of Phonon Anomalies and Superstructures in TTF-TCNQ. *J. Phys. Soc. Jpn.* **1976**, *41*, 2061–2071.

(S5) Pouget, J. P.; Khanna, S. K.; Denoyer, F.; Comès, R.; Garito, A. F.; Heeger, A. J. X Ray Observation of $2k_F$ and $4k_F$ Scatterings in Tetrathiafulvalene-Tetracyanoquinodimethane (TTF-TCNQ). *Phys. Rev. Lett.* **1976**, *37*, 437–440.

(S6) Comès, R.; Shirane, G.; Shapiro, S. M.; Garito, A. F.; Heeger, A. J. Elastic-Neutron-Scattering Study of the Phase Transitions in Tetrathiafulvalene-Tetracyanoquinodimethane (TTF-TCNQ). *Phys. Rev. B* **1976**, *14*, 2376–2383.

(S7) Comès, R.; Shapiro, S. M.; Shirane, G.; Garito, A. F.; Heeger, A. J. Neutron-Scattering Study of the 38- and 54-K Phase Transitions in Deuterated Tetrathiafulvalene-Tetracyanoquinodimethane (TTF-TCNQ). *Phys. Rev. Lett.* **1975**, *35*, 1518–1521.

(S8) Sutton, A. L.; Abrahams, B. F.; D'Alessandro, D. M.; Elliott, R. W.; Hudson, T. A.; Robson, R.; Usov, P. M. Structural and Optical Investigations of Charge Transfer Complexes Involving the F_4TCNQ Dianion. *CrystEngComm* **2014**, *16*, 5234–5243.

(S9) Konno, M.; Ishii, T.; Saito, Y. The Crystal Structures of the Low- and High-Temperature Modifications of Potassium 7,7,8,8-Tetracyanoquino dimethanide. *Acta Crystallogr. B* **1977**, *33*, 763–770.

(S10) Kistenmacher, T. J.; Phillips, T. E.; Cowan, D. O. The Crystal Structure of the 1:1 Radical Cation-Radical Anion Salt of 2,2'-Bis-l,3-dithiole (TTF) and 7,7,8,8-Tetracyanoquinodimethane (TCNQ). *Acta Crystallogr. B* **1974**, *30*, 763–768.

(S11) Tamura, S.; Kadoya, T.; Kawamoto, T.; Mori, T. Self-Contact Organic Transistors based on Tetramethyltetrathiafulvalene. *Appl. Phys. Lett.* **2013**, *102*, No. 063305.

(S12) Yamamoto, T.; Shinamura, S.; Miyazaki, E.; Takimiya, K. Three Structural Isomers of Dinaphthothieno[3,2-b]thiophenes: Elucidation of Physicochemical Properties, Crystal Structures, and Field-Effect Transistor Characteristics. *Bull. Chem. Soc. Jpn.* **2010**, *83*, 120-

130.

(S13) Kobayashi, H.; Kobayashi, N.; Hosoi, S.; Koshitani, N.; Murakami, D.; Shirasawa, R.; Kudo, Y.; Hobara, D.; Tokita, Y.; Itabashi, M. Hopping and Band Mobilities of Pentacene, Rubrene, and 2,7-Dioctyl[1]benzothieno[3,2-b][1]benzothiophene (C_8 -BTBT) from First Principle Calculations. *J. Chem. Phys*. **2013**, *139*, No. 014707.

(S14) Nishikawa, R.; Fan, S.; Kurashima, H.; Feng, J.; Kawamoto, T.; Le Gal, Y.; Lorcy, D.; Mori, T. n-Channel Transistor Properties of Fluoroalkyl and Trithion Form Birhodanines. *ACS Appl. Electron. Mater*. **2013**, *5*, 4254-4260.

(S15) Deng, W.-Q.; Goddard III, W. A. Predictions of Hole Mobilities in Oligoacene Organic Semiconductors from Quantum Mechanical Calculations, *J. Phys. Chem. B* **2004**, *108*, 8614-8621.

(S16) Hutchison, G. R.; Ratner, M. A.; Marks T. J. Hopping Transport in Conductive Heterocyclic Oligomers: Reorganization Energies and Substituent Effects. *J. Am. Chem. Soc*. **2005**, *127*, 2339-2350.

(S17) Oshi, R.; Abdalla, S.; Springborg, M. The Impact of Functionalization of Organic Semiconductors by Electron Donating Groups on the Reorganization Energy. *Eur. Phys. J. D* **2019**, *73*, No. 124.

(S18) Menard, E.; Podzorov, V.; Hur, S.-H.; Gaur, A.; Gershenson, M. E.; Rogers J. A. High-Performance n- and p-Type Single-Crystal Organic Transistors with Free-Space Gate Dielectrics. *Adv. Mater*. **2004**, *16*, 2097-2102.

(S19) Yamagishi, M.; Tominari, Y.; Uemura, T.; Takeya. J. Air-Stable n-Channel Single-Crystal Transistors with Negligible Threshold Gate Voltage. *Appl. Phys. Lett*. **2009**, *94*, No. 053305.

(S20) Takahashi, T.; Tamura, S.; Akiyama, Y.; Kadoya, T.; Kawamoto, T.; Mori, T. Organic Field-Effect Transistors Based on Small-Molecule Organic Semiconductors Evaporated under Low Vacuum. *Appl. Phys. Exp*. **2012**, *5*, No. 061601.

(S21) Kanazawa, K.; Bulgarevich, K.; Kawabata, K.; Takimiya, K. Uncovered Effects of thieno[2,3-*b*]thiophene Substructure in a Tetrathienoacene Backbone: Reorganization Energy and Intermolecular Interaction. *Chem. Mater*. **2023**, *35*, 280−288.

(S22) Sze, S. M. *Physics of Semiconductor Devices*, 2nd Ed. Wiley 1981, p 270-297.

(S23) Ishii, H.; Seki, K. Energy Level Alignment at Organic/Metal Interfaces Studied by UV Photoemission: Breakdown of Traditional Assumption of a Common Vacuum Level at the Interface. *IEEE Trans. Electron Devices* **1997**, *44*, 1295-1301.

(S24) Ishii, H.; Sugiyama, K.; Yoshimura, D.; Ito, E.; Ouchi, Y.; Seki, K. Energy-Level Alignment at Model Interfaces of Organic Electroluminescent Devices Studied by UV Photoemission: Trend in the Deviation from the Traditional Way of Estimating the Interfacial Electronic Structures. *IEEE J. Sel. Top. Quantum Electron*. **1998**, *4*, 24-33.

(S25) Hill, I. G.; Kahn, A. Interface Electronic Properties of Organic Molecular Semiconductors. in *Organic Light-Emitting Materials and Devices II. Proceedings of SPIE*, ed. by Z. H. Kafafi, **1998**, *3476*, 168.

(S26) Di, C.; Yu, G.; Liu, Y.; Xu, X.; Wei, D.; Song, Y.; Sun, Y.; Wang, Y.; Zhu, D.; Liu, J.; Liu, X.; Wu, D. High-Performance Low-Cost Organic Field-Effect Transistors with Chemically Modified Bottom Electrodes. *J. Am. Chem. Soc.* **2006**, *128*, 16418-16419.

(S27) Yu, Y.; Kanno, M.; Wada, H.; Bando, Y.; Ashizawa, M.; Tanioka, A.; Mori, T. Enhanced Performance of Bottom-Contact Organic Field-Effect Transistors with $M(DMDCNQI)_2$ Buffer Layers. *Physica* **2010**, *405*, S378-380.

(S28) Ohta, E.; Nagano, T.; Ochi, k.; Kubozono Y.; Shikoh, E.; Fujiwara, A. Variation of Output Properties of Perylene Field-Effect Transistors by Work Function of Source/Drain Electrodes. *Appl. Phys. Lett.* **2006**, *89*, No. 053508.

(S29) Wada, H.; Shibata, K.; Bando, Y.; Mori, T. Contact Resistance and Electrode Material Dependence of Air-Stable n-Channel Organic Field-Effect Transistors Using Dimethyldicyanoquinonediimine (DMDCNQI). *J. Mater. Chem.* **2008**, *18*, 4165-4171.
